# Design, Synthesis, and Action Mechanism of 1,3-Benzodioxole Derivatives as Potent Auxin Receptor Agonists and Root Growth Promoters

**DOI:** 10.3389/fpls.2022.902902

**Published:** 2022-06-10

**Authors:** Zhikun Yang, Jiahui Xu, Lin Du, Jiaming Yin, Zhao Wang, Fei Yi, Liusheng Duan, Zhaohu Li, Baomin Wang, Kai Shu, Weiming Tan

**Affiliations:** ^1^College of Agronomy and Biotechnology, China Agricultural University, Beijing, China; ^2^College of Pharmaceutical Science & Green Pharmaceutical Collaborative Innovation Center of Yangtze River Del-ta Region, Zhejiang University of Technology, Hangzhou, China; ^3^School of Ecology and Environment, Northwestern Polytechnical University, Xi’an, China

**Keywords:** virtual screening, 1, 3-benzodioxole derivatives, TIR1, auxin response, root growth regulator

## Abstract

Deeper and longer roots allow crops to survive and flourish, but our understanding of the plant growth regulators promoting root system establishment is limited. Here, we report that, a novel auxin receptor agonist, named K-10, had a remarkable promotive effect on root growth in both *Arabidopsis thaliana* and *Oryza sativa* through the enhancement of root-related signaling responses. Using computer-aided drug discovery approaches, we developed potent lead compound by screening artificial chemicals on the basis of the auxin receptor TIR1 (Transport Inhibitor Response 1), and a series of N-(benzo[d] [1,3] dioxol-5-yl)-2-(one-benzylthio) acetamides, K-1 to K-22, were designed and synthesized. The results of bioassay showed that K-10 exhibited an excellent root growth-promoting activity far exceeding that of NAA (1-naphthylacetic acid). A further morphological investigation of the auxin related mutants (*yucQ*, *tir1*) revealed that K-10 had auxin-like physiological functions and was recognized by TIR1, and K-10 significantly enhanced auxin response reporter’s (*DR5:GUS*) transcriptional activity. Consistently, transcriptome analysis showed that K-10 induced a common transcriptional response with auxin and down-regulated the expression of root growth-inhibiting genes. Further molecular docking analysis revealed that K-10 had a stronger binding ability with TIR1 than NAA. These results indicated that this class of derivatives could be a promising scaffold for the discovery and development of novel auxin receptor agonists, and the employment of K-10 may be effective for enhancing root growth and crop production.

## Introduction

Root systems are significant plant components that effect performance and yield. Roots not only anchor plants in the soil, but also enable water uptake and nutrient acquisition (Coudert et al., 2010). To improve crop yields, the production of deeper and wider roots has been a research focus (Meng et al., 2019; Motte et al., 2019; Severini et al., 2020). In agricultural production, the perfect root phenotypes are shaped through the development of new varieties (Schneider et al., 2020), strengthening of nutrition management (Hirte et al., 2018; Wang H. et al., 2019) and the use of phytohormone derivatives as plant growth regulators (Frick and Strader, 2018; Madan and Sangeeta, 2018). Unfortunately, the development of new varieties is time-consuming, and nutrition management requires significant resource support and is difficult to maintain. The plant growth regulator approach is an efficient and low support strategy.

Root growth and development are regulated by multiple phytohormones, among which auxins play major roles (Ishimaru et al., 2018). Owing to the unstable chemical properties of natural auxins, such as indole-3-acetic acid (IAA), indol-3-butyric acid (IBA), phenylacetic acid (PAA), and 4-chloroindole-acetic acid (4-Cl-IAA), artificial synthetic auxins, such as 1-naphthylacetic acid (NAA), 2, 4-dichlorophenoxyacetic acid (2,4-D), 4-Chlorophenoxyacetic acid (4-CPA), and 2-methyl-4-chlorophenoxyacetic acid (MCPA), have been employed as plant growth regulators in agricultural production. However, these commercial auxin-like chemicals have limited promotive effects on root growth, especially the elongation of primary roots (Chi-Linh et al., 2016; Guan et al., 2019), and thus, treated plants do not have roots that meet the requirements for desired root phenotypes. Thus, there is an urgent need for plant growth regulators that have satisfactory root-promotive effects.

In the past decades, most studies focused on the action mechanisms of auxins. Auxins are recognized through the receptor transport inhibitor response 1 (TIR1)/auxin signaling F-box protein and then, they affect the association between auxin/IAA proteins and auxin response factor proteins, which activate or repress downstream gene transcription and mediate auxin responses (Gray et al., 2001; Richard et al., 2002; Nihal et al., 2005; Stefan and Ottoline, 2005). The X-ray crystal structure of the TIR1-apoptosis signal-regulating kinase 1 complex and mechanisms of auxin perception have been reported (Tan et al., 2007), providing a bioinformatics basis for the computer-aided drug discovery of auxin agonists. With the rapid development of chemicobiology and computer-aided technologies, successful cases of computer-aided drug discovery continue to emerge (William, 2009; Lavecchia and Giovanni, 2013; Cavasotto et al., 2019). For example, opabactin (Vaidya et al., 2019) and AMF4 (Cao et al., 2013, 2017), the outstanding abscisic acid (ABA) receptor agonists, were obtained using computer-aided screening and computer-guided optimization, providing a potential approach for drought resistance in agricultural production systems. However, few studies have focused on the discovery of auxin receptor agonists acting as root growth promoters.

In our research, the lead compound *N*-(benzo[*d*][1,3]dioxol-5-yl)-2-((4-chlorobenzyl) thio) acetamide, named HTS05309, was screened on the basis of the pharmacophore model and the auxin receptor TIR1. A series of *N*-(benzo[*d*][1,3]dioxol-5-yl)-2-(one-benzylthio) acetamide compounds were designed using empirical modifications, synthesized in a three-step reaction and characterized by ^1^H NMR, ^13^C NMR and high-resolution mass spectrometry. Subsequently, the biological activities of the target compounds were evaluated by calculating the root-promotive effects in *Arabidopsis thaliana* and *Oryza sativa*. Further, the root morphologies were observed and recorded by scanning and staining, and then the structure–activity relationships (SAR) were investigated. Furthermore, the auxin-like physiological functions of K-10 were studied by investigating the responses of mutant (*yucQ*), an *Arabidopsis* auxin reporter (*DR5:GUS*) and endogenous IAA concentrations in *O. sativa*, and the recognition of K-10 was studied by investigating the phenotypes of a *tir1* mutant. Additionally, a genome-wide transcriptome analysis by RNA sequencing of *O. sativa* roots was performed to further verify the auxin-like functions of K-10 and revealed the mechanism of its root growth-promoting activity. Finally, molecular docking was performed to investigate the binding mode of K-10 with the TIR1.

## Results and Discussion

### Virtual Screening and Artificial Synthesis of Potential Auxin Receptor Agonists

**FIGURE 1:**
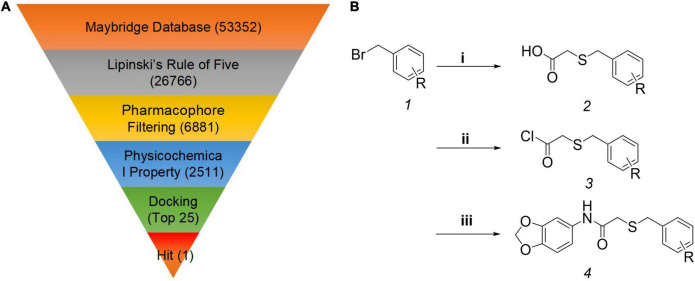
Computer-aided drug design and the synthesis of target compounds K-1 to K-22. **(A)** The virtual screening workflow. **(B)** The synthesis route of the target compounds K-1 to K-22*. *Reagent and conditions: (i) thioglycolic acid, NaOH, EtOH/H_2_O, 78°C, 3 h; (ii) oxalyl chloride, dichloromethane, 0°C to room temperature, 1.5 h; (iii) benzo[*d*][1,3]dioxol-5-amine, triethylamine, dichloromethane, 0°C to rt, 1.5 h. R = 2-CF_3_, 3-CF_3_, 4-CF_3_, 2-F, 3-F, 4-F, 2-Cl, 3-Cl, 2-Br, 3-Br, 4-Br, 2-I, 3-I, 4-I, H, 3-CH_3_, 3-OCH_3_, 2,3-di-Cl, 2,5-di-Cl, 2,6-di-Cl, 3,4-di-Cl, 3,5-di-Br.

To identify the novel auxin agonists, the Maybridge database containing 53,352 small molecules, was selected for virtual screening using Molecular Operation Environment software. On the basis of herbicide-likeness, 26,766 small molecules were selected using Lipinski’s Rule of Five (Lipinski et al., 1996). To further improve the screening accuracy and efficiency, the pharmacophore models were established, and the pharmacophore models with the highest overlap (5.60), containing hydrophobic centroid/aromatic centroid, hydrogen-bond acceptor and hydrophobic centroid, was selected (Supplementary Figure 1). Through pharmacophore screening, 6,881 molecules were obtained. To further reduce the number of small molecules, physicochemical properties were applied as filters, resulting in 2,511 molecules (Rao et al., 2015; Zhang Y. et al., 2018). Finally, molecular docking was performed between the 2,511 molecules and the auxin receptor protein TIR1, and the 25 compounds that scored in the top 1% were selected (Figure 1A). Intriguingly, HTS05309, which was found to possess bioactivity in our previous study (Wang J. et al., 2019), was one of these 25 compounds. Previous studies demonstrated that some molecules tend to have multiple targets (Klessig et al., 2016), HTS05309 was obtained by different virtual screening based on receptors GID1 or TIR1, which indicated the molecule HTS05309 might exhibit the ability to bind with TIR1 and GID1. Ultimately, HTS05309 was selected as the lead compound because of its excellent properties, such as easy chemical synthesis, low cost and good solubility.

As we know, plant growth regulators have been widely used in field crop research and have made great contributions to agricultural production (Motyka, 2005). Researchers have developed various plant growth regulators by virtual screening or/and the empirical modifications, such as uniconazole, which adjusts plant height to resist lodging (Ahmad et al., 2019), thidiazuron, which sheds plant leaves for mechanical harvest to reduce labor (Suttle, 1985; Ledbetter and Preece, 2004), and opabactin (Vaidya et al., 2019), which mitigates the effects of drought, reducing risks to crop yields. Combining the chemical structure of HTS05309 with the empirical modifications, 22 novel *N*-(benzo[*d*] [1,3] dioxol-5-yl)-2-(one-benzylthio) acetamide compounds were designed and named K-1 to K-22. All target compounds were synthesized in three steps (Figure 1B). Initially, the secondary intermediates were prepared through a substitution reaction between substituted benzyl bromide and thioglycolic acid, and the crude products were directly used in the next reaction without purification. Subsequently, the carboxyl of the secondary intermediates was transformed into acid chloride, which was convenient for reactions with organic amine. Then, target products were isolated using column chromatograph, with yields of 37–90%. Obviously, target compounds were synthesized in three-step reactions with high yields, and they were conducive to production and agricultural applications.

### The Root Growth-Promoting Activity and Structure-Activity Relationship Study

**FIGURE 2:**
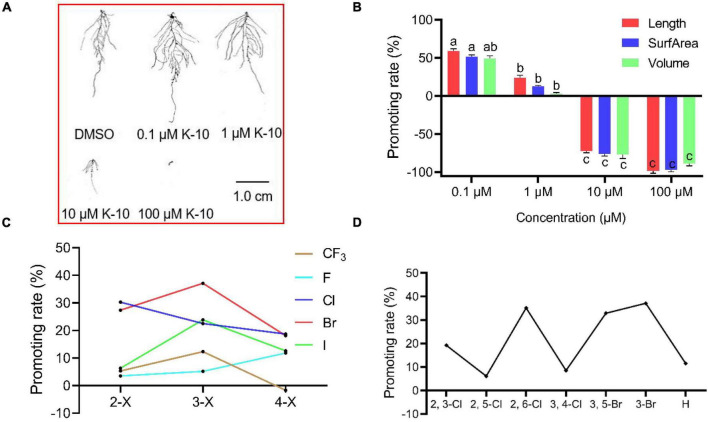
Target compounds promote the root growth of 14-day-old *Arabidopsis* (Col-0) and a structure–activity relationship analysis. **(A)** Root scan images of 14-day-old *Arabidopsis* treated with K-10. **(B)** Effects of K-10 on the root growth of 14-day-old *Arabidopsis*. **(C)** Parallel activity contrast studies of target compounds having different substituted benzene rings. **(D)** Parallel activity contrast studies of target compounds having different multi-substituted benzene rings. Bars represent SEs of averages (*n* = 3).

To explore the root growth-promoting bioactivities of the target compounds, their preliminary bioactivities were investigated in *Arabidopsis*. Most compounds displayed a significant promotive effect on *Arabidopsis* primary root growth at a dose of 0.1 μM, whereas some compounds displayed inhibitory effects on primary root growth at a dose of 1 μM (Table 1). In particular, K-10 displayed an outstanding effect, with a 37.1% promotive rate on *Arabidopsis* primary roots at a dose of 0.1 μM, which compared favorably with HTS05309 (18.7%) and NAA (–36.9%). Furthermore, a root structure scan analysis showed that K-10 significantly promoted root growth, including primary root lengths and root numbers, at doses of 0.1 and 1 μM, after which the root lengths, surface areas and volumes increased more than 50% compared with controls (Figures 2A,B).

**TABLE 1:** The growth promoting rate of target compounds, HTS05309 and NAA on the primary root of 7-day-old *Arabidopsis* (Col-0) and 7-day-old *Oryza sativa* (Nihonbare).

Compd	R	*Arabidopsis* (% Promotion)		*Oryza sativa* (% Promotion)	
			
		0.1 μM	1 μM	1/0.005 μM	1/0.05 μM
K-1	2-CF_3_	5.3 ± 0.1	7.8 ± 2.1	5.8 ± 0.2	–12.3 ± 0.7
K-2	3-CF_3_	12.3 ± 1.5	–6.8 ± 0.2	1.0 ± 0.1	13.5 ± 1.0
K-3	4-CF_3_	–1.7 ± 0.2	–26.20.6	10.0 ± 0.7	–9.5 ± 0.7
K-4	2-F	3.5 ± 0.1	–31.4 ± 0.4	14.4 ± 1.0	40.7 ± 1.2
K-5	3-F	5.2 ± 0.4	–18 ± 2.1	22.2 ± 2.0	–6.4 ± 0.5
K-6	4-F	11.8 ± 0.3	–27 ± 1.9	11.0 ± 0.9	38.4 ± 1.0
K-7	2-Cl	30.3 ± 0.7	–1.5 ± 0.1	21.4 ± 3.0	49.1 ± 1.3
K-8	3-Cl	22.5 ± 1.7	7.8 ± 0.7	8.7 ± 1.8	–9.9 ± 0.5
HTS05309	4-Cl	18.7 ± 1.5	7.3 ± 0.5	10.4 ± 1.4	–0.6 ± 0.1
K-9	2-Br	27.4 ± 0.8	5.5 ± 0.2	14.6 ± 0.6	41.8 ± 2.0
K-10	3-Br	37.1 ± 2.0	8.6 ± 0.9	10.7 ± 1.2	17.9 ± 1.6
K-11	4-Br	18.1 ± 0.5	1.9 ± 0.1	34.4 ± 2.0	65.1 ± 2.0
K-12	2-I	6.3 ± 0.4	2.2 ± 0.2	18.8 ± 1.2	54.0 ± 3.0
K-13	3-I	23.9 ± 0.7	–23.4 ± 0.5	5.4 ± 1.1	–9.9 ± 2.1
K-14	4-I	12.6 ± 1.3	–12.9 ± 1.1	2.0 ± 0.1	32.1 ± 1.0
K-15	H	11.5 ± 0.4	–14.4 ± 0.9	6.4 ± 0.5	39.3 ± 2.0
K-16	3-CH_3_	2.1 ± 0.3	–4.5 ± 0.3	15.8 ± 2.0	33.6 ± 1.0
K-17	3-OCH_3_	8.3 ± 0.1	–15.8 ± 0.8	5.6 ± 0.4	31.6 ± 2.1
K-18	2,3-di-Cl	19.2 ± 1.1	–25.6 ± 1.2	9.2 ± 0.7	46.7 ± 1.8
K-19	2,5-di-Cl	6.1 ± 0.5	–15.3 ± 3.1	2.1 ± 0.2	–7.4 ± 1.1
K-20	2,6-di-Cl	35.1 ± 2.3	17.9 ± 2.9	2.6 ± 0.2	–4.4 ± 0.4
K-21	2,3-di-Cl	8.5 ± 1.2	5.1 ± 0.3	11.8 ± 1.0	48.5 ± 3.0
K-22	3,4-di-Cl	32.9 ± 0.8	16.2 ± 0.9	21.0 ± 2.0	5.8 ± 0.3
NAA	3,5-di-Br	–36.9 ± 1.6	–87 ± 0.7	8.0 ± 0.9	–7.8 ± 0.7

*Data are the mean of three-independent determinations of triplicate samples.*

To analyze the SAR, parallel activity contrast studies of target compounds containing different substituted benzene rings were performed based on the results of the *Arabidopsis* primary root. Obviously, the introduction of electron-withdrawing groups (3-Cl, 3-Br, 3-I) was conducive to enhancing the bioactivity of target compounds, while the electron-donating groups (3-CH_3_, 3-OCH_3_) leaded to a decrease in activity. Moreover, the introduction of chlorine and bromine atoms brings a higher activity than compounds containing fluorine and iodine atoms, K-10 having a *meta*-bromophenyl group displayed a greater promotive activity than other target compounds, which indicated that *meta*-bromine introduced into the benzene ring was beneficial in improving the promotive activity (Figure 2C). Comparisons of target compounds substituted with multiple halogens on the benzene ring (compounds K-18 to K-22) revealed that compounds K-18, K-20 and K-22 had better promotive activities than compound K-15, but not as good as K-10, which indicated that the introduction of multiple electron-withdrawing groups into the appropriate position on the benzene ring was beneficial in improving the promotive activity (Figure 2D). Thus, empirical modification was a successful strategy that led to optimized chemical structure of lead HTS05309, resulting in the discovery of K-10.

**FIGURE 3:**
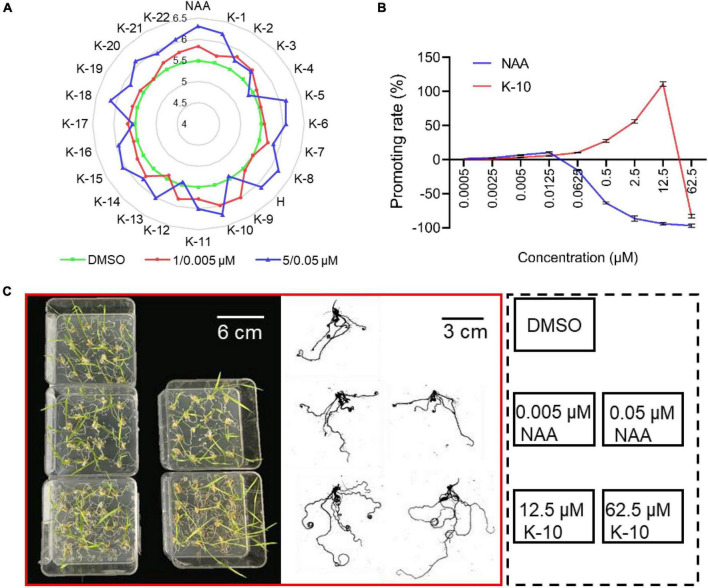
Target compounds promote the root growth of *Oryza sativa* (Nihonbare). **(A)** Root numbers of 7-day-old *Oryza sativa* treated with target compounds, H (HTS05309) and NAA. **(B)** Effects of K-10 and NAA on the primary root growth of 7-day-old *O. sativa*. **(C)** Root growth performance of 12-day-old *O. sativa* seedlings treated with K-10 and NAA. **(D)** Effects of K-10 and NAA on the root growth of 12-day-old *O. sativa*. Bars represent SEs of averages (*n* = 3).

Subsequently, the monocotyledonous model crop *O. sativa* was used to investigate the application and action mechanisms of target compounds. The preliminary bioactivities of target compounds were examined on *O. sativa* roots, including the primary root length and total root numbers. Most of the target compounds displayed a significant promotive effect on primary root elongation and the generation of lateral roots at a dose of 1 μM, and the promotive activity increased when the dose was increased to 5 μM (Table 1 and Figure 3A). In particular, K-10 exhibited an excellent activity, resulting in promotive rates of 34.4 and 65.1% on *O. sativa* primary root elongation at doses of 1 and 5 μM, respectively, which were greater than those of HTS05309 (14.6 and 41.8%) at the same concentrations, respectively, and the reference NAA (5.8 and –12.3%) at doses of 0.005 and 0.05 μM, respectively (Table 1). Notably, due to species variability, some compounds tend to show varying degrees of bioactivity on different plants, but K-10 stood out from all target compounds, showed the best bioactivity on both monocotyledonous and dicotyledonous plants, perhaps suggesting that the action mode of K-10 is universal in plants.

To further compare the bioactivities of K-10 and NAA, dose curves of their promotive effects on the elongation of *O. sativa* primary roots were constructed (Figure 3B). K-10 and NAA displayed promotive activities on primary root elongation at lower doses, but the increases were not significant. The promotive effect of K-10 on the elongation of *O. sativa* primary roots far exceeded that of NAA as the treatment concentration increased, especially in K-10 producing a 110.8% promotive rate at 12.5 μM, which was in line with our original vision. Furthermore, the root morphology of 12-day-old *O. sativa* seedlings treated with K-10 was investigated (Figures 3C,D). K-10 significantly promoted root growth, including root length, surface area and volume, to a greater extent than NAA. Thus, K-10 was a more potential root growth promoter than NAA, which could make up for the agricultural deficiency that auxins displayed weak promotive effects on root elongation at low concentrations, but strongly inhibitory effects on root growth at high concentrations (Ledbetter and Preece, 2004).

**FIGURE 4:**
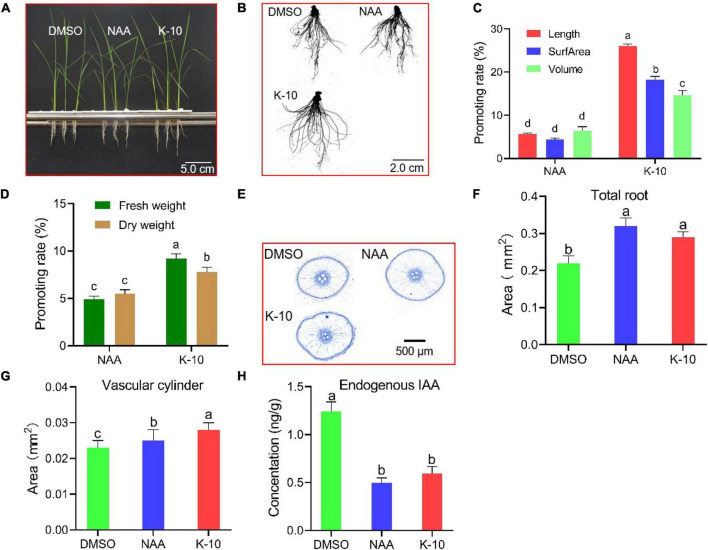
Modifications in *Oryza sativa* (Nihonbare) seedling root development after being treated with K-10. **(A–C)** Growth characteristics of 25-day-old *O. sativa* treated with DMSO (solution), 0.005 μM NAA and 12.5 μM K-10. **(D)** Effects of K-10 and NAA on dry matter accumulations of 25-day-old *O. sativa* roots. **(E)** Representative cross-sections of *O. sativa* roots stained with toluidine blue O. **(F)** Total root areas. **(G)** Vascular cylinder areas. **(H)** Endogenous hormone (IAA) concentrations in *O. sativa* roots. Bars represent SEs of averages (*n* = 3).

For root sampling, and both transcriptomics and metabolomics analyses, *O. sativa* seedlings were hydroponically cultured. The growth characteristics of 25-day-old *O. sativa* seedlings at 5 days after treatment with K-10 and NAA were recorded. K-10 displayed a more significant promotive activity on root growth and dry matter accumulation than NAA (Figures 4A–D). Additionally, the promotive activities of K-10 and NAA decreased compared with the results on an agar medium, which was attributed to the older seedlings displaying a weaker sensitivity to auxin (Figure 3D). In addition, to observe the effects of K-10 on the growth and development of *O. sativa* seedlings, sliced cross-sections of *O. sativa* embryonic adventitious roots (maturity area, 1-1.5 cm) were recorded. K-10 increased the *O. sativa* seedling embryonic adventitious root cross-sectional area (Figures 4E,F) and vascular cylinder area (Figures 4E,G), which was consistent with auxin’s promotion in differentiation of vascular tissue. To determine that the increased auxin activity and promoted root growth in response to K-10 was not the result of an elevated auxin concentration, endogenous auxin (IAA) concentrations were measured in *O. sativa* roots. The free IAA concentrations significantly decreased in *O. sativa* roots treated with NAA and K-10, which indicated that K-10, like NAA, possesses an *in vivo* auxin-like bioactivity (Figure 4H).

### Biological Study in Action Mechanism of K-10

**FIGURE 5:**
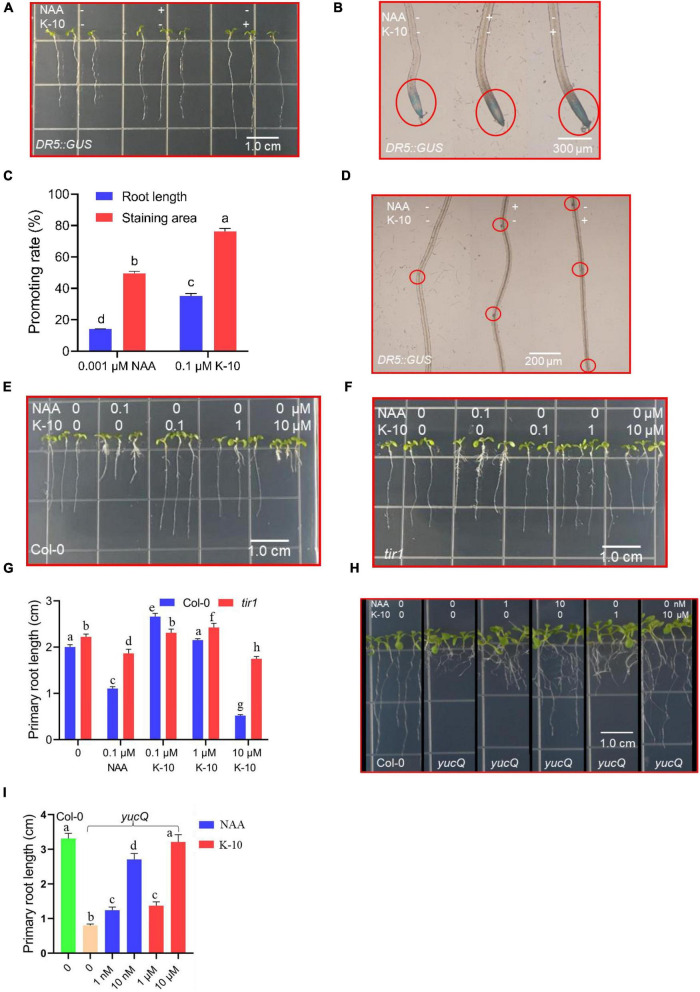
Modifications in *Arabidopsis* (*DR5:GUS; tir1; yucQ*) seedling root development after being treated with K-10. **(A)** Growth characteristics of 5-day-old *Arabidopsis* (*DR5:GUS*) treated with DMSO (solution), 0.001 μM NAA or 0.1 μM K-10. **(B)** Histochemical *GUS* analysis of roots from *DR5:GUS* seedlings treated with DMSO, 0.001 μM NAA or 0.1 μM K-10. **(C)** Quantitative *GUS* and root length analyses. **(D)** Numbers of primordia in roots of *DR5:GUS* seedlings treated with DMSO, 0.001 μM NAA or 0.1 μM K-10. **(E,F,H)** Growth characteristics of 7-day-old *Arabidopsis* (Col-0, *tir1*, *yucQ*) treated with DMSO, NAA or K-10. **(G,I)** Effects of K-10 and NAA on the primary root growth of 7-day-old *Arabidopsis* (Col-0, *tir1*, *yucQ*). Bars represent SEs of averages (*n* = 3).

Auxins play decisive roles in the processes of root growth and development (Matthes et al., 2019; Vain et al., 2019; Zha et al., 2019), and K-10 showed a significant auxin-like activity as assessed by phenotypic observations of *Arabidopsis* and *O. sativa*. The genetic approach, an accurate and efficient *in vivo* biological evaluation, was used to explore the action modes of the novel compounds, and the responses of the reporter *DR5:GUS* and the phenotypes of *tir1*, *yucQ* mutants, which are used in the auxin-related research (Oono et al., 2003; Chen et al., 2014; Taisuke et al., 2015), provide powerful tools for certifying the auxin-like function of K-10. Therefore, we studied its effects on the auxin-mediated transcriptional regulation using the reporter *DR5:GUS* in *Arabidopsis* primary roots. The activity of *DR5* in primary roots increased in response to NAA and K-10, suggesting that K-10 effectively induced the auxin response, like NAA (Figures 5A,B). The *GUS* activity was greater in response to K-10 than in response to NAA (Figure 5C). In addition, the root primordia numbers of *Arabidopsis* treated with NAA, K-10, DMSO were 2.6, 3.2 and 1.5, respectively, indicating that K-10 exhibited a better activity in promotion in the differentiation of root primordia (Figure 5D). Furthermore, the primary roots of a *tir1* mutant were longer than those of *Arabidopsis* (Col-0) in response to NAA, suggesting that the sensitivity of the *tir1* loss-of-function mutant to NAA decreased (Figures 5E–G). Consistent with the biological phenotypes of NAA and K-10 responses, the sensitivity of the *tir1* mutant to K-10 also decreased, which indicated that the *tir1* mutant lost the ability to recognize K-10. In addition, the roots of *yucQ* mutant were restored to growth by K-10 and NAA, suggesting that K-10 exhibited a significant auxin-like activity (Figures 5H,I).

### Transcriptome Analysis

**FIGURE 6:**
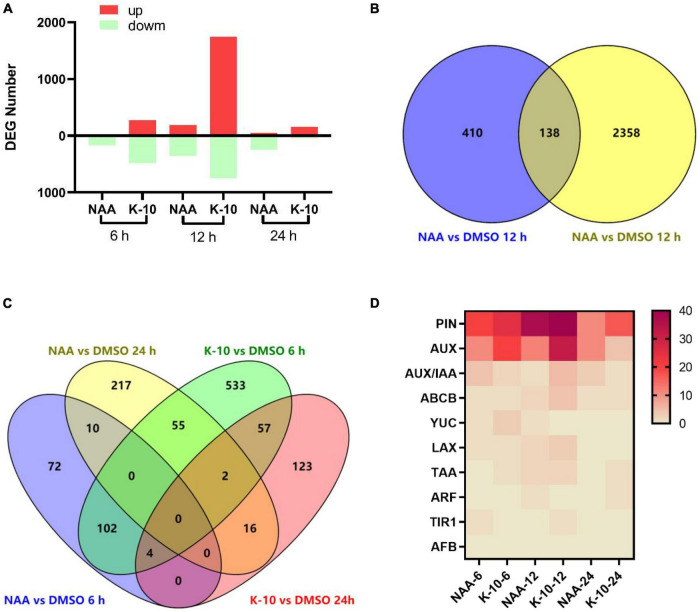
K-10 regulates auxin-responsive and root-related genes in *Oryza sativa* (Nihonbare) roots. **(A)** DEG numbers resulting from K-10 and NAA priming that impact *Oryza sativa* root development. **(B,C)** Overlap of NAA- and K-10-induced DEGs. **(D)** Heat map of Auxin-related DEG numbers resulting from NAA and K-10 priming that impact *O. sativa* root development.

To explore the nature of the functions and the excellent root growth-promoting activity of K-10 at the molecular level, which leads to the observed growth responses, we performed a genome-wide transcriptome analysis using RNA sequencing. The correlation analysis among 18 samples revealed that the biologically repeated samples had good repeatability and the results could be used for further statistical analyses (Supplementary Figure 2). When plants were exposed to NAA for 6, 12, and 24 h, 188, 548, and 300 genes, respectively, were significantly affected, whereas K-10 resulted in the 753, 2,496, and 202 DEGs, respectively, which demonstrated that K-10 exposure resulted in more genetic responses more quickly than NAA (Figure 6A). Consistent with the hypothesis that K-10 is an auxin agonist, there was overlap in the gene pools between the K-10 and NAA treatments (Figures 6B,C). In total, 34% (138 of 410) of the DEGs expressed in response to the 12-h NAA treatment were also affected by the 12-h K-10 treatment, and 19% (57 of 300) of the DEGs expressed in response to the 24-h NAA treatments were also affected by the 12-h K-10 treatment. A gene ontology enrichment analysis revealed that the processes (catalytic activity, binding, cellular process, cell part, membrane part, metabolic process, etc.) were highly enriched with K-10- specific DEGs at 6, 12, and 24 h after treatment, which is consistent with the responses caused by NAA at 6, 12, and 24 h after treatment (Supplementary Figure 3).

In the present investigation, the auxin biosynthesis, transport and signaling pathways were revealed at the molecular level, providing an essential reference for exploring the nature of new auxin agonists (Kalve et al., 2020). The genes ARABIDOPSIS–YUCCA (*TAA*–*YUC*), PINFORMED (*PIN*), PIN-LIKES (*PILS*), ATP-binding cassette family B (*ABCB*)-P-glycoprotein (*PGP*), AUXIN1/LIKE-AUX (*AUX*/*LAX*), TRANSPORTINHIBITOR RESPONSE1/AUXIN-RELATED F-BOX (*TIR1*/*AFB*), AUXIN RESPONSE FACTORs (*ARFs*), and TOPLESS (*TPL*) play important roles in the pathways of auxin biosynthesis, transport and signaling (Grones and Friml, 2015; Harrison, 2017; Růžička and Hejtko, 2017; Leyser, 2018; Zhang T. et al., 2018). To further verify that the action mechanism of K-10 was related to the auxin pathway, the genes related to auxin biosynthesis, transport and signaling were collected and compared with the DEGs expressed in response to the NAA and K-10 treatments. As expected, there was a great deal of overlap between the auxin-synthesis, transport and signaling genes and the DEGs expressed in response to the NAA treatment (Figure 6D). Consistent with the NAA treatment, the DEGs expressed in response to the K-10 treatment also overlapped with the auxin-synthesis, transport and signaling genes. This explained the auxin-like functions of K-10. In particular, K-10, like NAA, elicited the responses of *TIR1*-related genes, but not auxin signaling F-box-related genes, which is consistent with the phenotype of the *Arabidopsis tir1* mutant.

Furthermore, *GH3*, *YUC*, *LAX*, *Aux/IAA*, *SAUR, TIR1* were chosen to explore the expression levels of genes in auxin biosynthesis, transport and signal transduction. As shown in Supplementary Figure 4, K-10 down-regulated the expression of multiple *GH3*- and *YUC*-related genes at 6, 12, and 24 h, such as Os11g0287100, Os11g0186500, and Os05g0500900, suggesting that K-10 inhibited the biosynthesis of IAA, which was consistent with the decrease in endogenous IAA content. Besides, K-10 up-regulated the expression of multiple *LAX*-, *Aux/IAA*-, *SAUR*-, and *TIR1*-related genes at 6, 12, and 24 h, such as Os01g0120400, Os03g0244600, Os05g0559400, Os02g0164900, and so on, indicating that auxin transport and signal transduction were still being carried out efficiently, which may be attributed to the auxin-like physiological functions of K-10 and was consistent with the enhanced auxin response.

**FIGURE 7:**
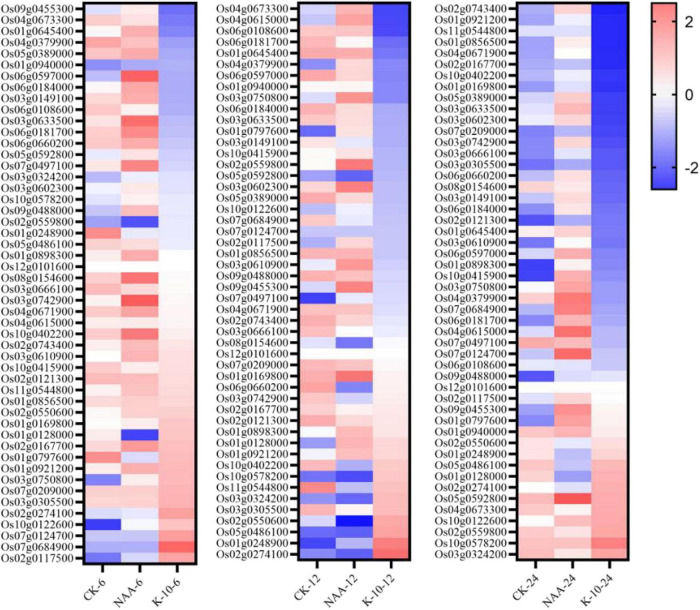
Heat map of root-related gene expression levels in *Oryza sativa* (Nihonbare) roots treated with K-10 or NAA at 6, 12, and 24 h.

To further explore the nature of the excellent root growth-promoting activity of K-10, 50 reported *O. sativa* root-related genes were investigated. Half of the 50 genes involved in multiple biological processes were associated with auxin signal transduction, indicating that auxin was very important to the growth and development of *O. sativa* roots (Meng et al., 2019). Interestingly, 42 of these 50 genes exhibited an inhibitory effect on the elongation of primary roots and the generation of lateral roots. Thus, down-regulating these 42 genes might be a successful strategy for promoting root growth. When plants were exposed to NAA for 6, 12, and 24 h, 12, 29, and 12 genes, respectively, were significantly downregulated, whereas exposure to K-10 resulted in 27, 33, and 30 genes, respectively, being downregulated (Figure 7). Thus, K-10 resulted in the down-regulated expression of more reported root-inhibiting genes than NAA, which may explain why K-10 displayed a greater promotive effect on *Oryza sativa* root growth.

### Molecular Docking

**FIGURE 8:**
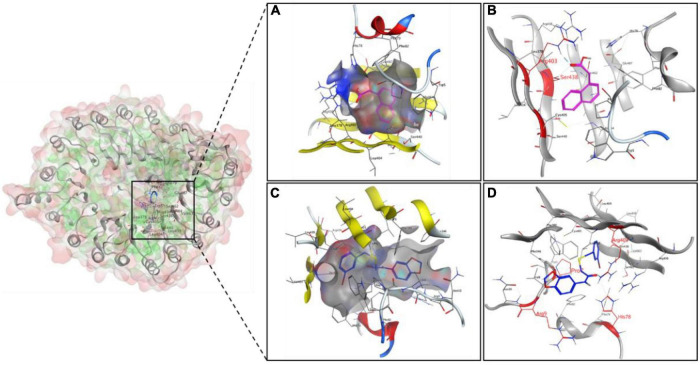
The interaction diagram of the ligands NAA **(A,B)** and K-10 **(C,D)** with auxin receptor TIR1. The cyan dotted lines represent hydrogen bond interactions.

To explore the binding modes of K-10 with auxin receptor TIR1, a molecular docking analysis was performed. The lowest binding energy for the docked conformations was chosen from 200 conformations as the representative binding energy and modes for the corresponding K-10 and NAA (Supplementary Figure 5). The binding energy of K-10 (–8.62 kJ mol^–1^) was lower than that of NAA (–7.67 kJ mol^–1^), indicating that K-10 had a greater affinity for TIR1. K-10 and NAA were both well wrapped by the active pocket. The oxygens, sulfurs of K-10 formed H-bond interactions with the residues His A78, Arg A403, and Arg B9 and the residue Pro B7, respectively, while the oxygens of NAA formed H-bond interactions with the residues Arg A403 and Ser A438, indicating that there were more H-bond interactions between K-10 and the receptor (Figure 8). Thus, K-10 possessed a stronger binding ability with receptor TIR1 than NAA, which might result in the excellent promotive effects on root growth and development.

## Materials and Methods

### Equipment and Materials

The virtual screening and molecular docking were performed by software SYBYL (Tripos, United States), Autodock (Scripps, United States) and Molecular Operate Environment (Chemical Computing Group Inc, Canada). All commercially available reagents were purchased from J&K Chemicals (Beijing, China), Tongguang (Beijing, China) without any extra treatment. The reaction progress was monitored by thin-layer chromatography on silica gel GF-254 with ultraviolet detection, and column chromatography was conducted on a silica gel plug (200-300 mesh). ^1^H NMR and ^13^C NMR spectra were recorded utilizing a Bruker AVANCE DPX300 spectrometer at 300 MHz in CDCl_3_ or DMSO-*d*_6_ solution with tetramethylsilane as the internal standard. HRMS (high-resolution mass spectra) data were obtained with an Agilent 6520 Accurate-Mass-Q-TOF LC/MS system, equipped with an electrospray ionization (ESI) source in the positive ionization mode. The melting points were obtained with a X-4 series microscope melting point apparatus (Shanghai, Jingmi) and were uncorrected. The plants *Arabidopsis* Columbia (Col-0) and *Oryza sativa* seeds (Nihonbare) were provided by the state key lab of plant physiology and biochemistry, China Agricultural University (Beijing, China), and all transgenic and mutants lines (*DR5:GUS*; *tir1*; *yucQ*) were derived from *Arabidopsis* Columbia (Col-0) and conserved by the key laboratory of crop ecophysiology and farming system, Sichuan Agricultural University (Chengdu, China). The *A. thaliana* and *O. sativa* growth data were obtained using ImageJ^1^ and WinRHIZO root scanner. The root morphology was observed and recorded with scanning microscope (BZ-X710, Keyence, Japan). The endogenous IAA concentrations were measured using a high-performance liquid chromatography-mass spectrometry (Acquity, Waters, United States; Q Exactive, Thermo, United States). The data of transcriptomics were analyzed on the free online platform of Majorbio Cloud Platform.^2^

### Virtual Screening

Molecules were obtained from the Maybridge portfolio^3^ and filtered by Molecular Operation Environment software. Initially, molecules were protonated, add explicit hydrogens and rebuild 3D using the Molecule module. And then, the Maybridge database was filtered using Lipinski’s Rule of Five (MW, <500, molecular weight; HBD, <5, number of hydrogen-bond donors; HBA, <5, number of hydrogen-bond acceptors; Log *P*, <5, Log octanal/water partition coefficient; ROB, < 10, number of rotatable bonds). Subsequently, the pharmacophore models were established used MOE pharmacophore module based on auxins (IAA, IBA, 4-Cl-IAA, PAA) and auxin-like compounds (NAA, 4-CPA, MCPA, 2,4-D), and the resultant molecules were screened against the pharmacophore models and based on physicochemical property (MW, 300-450; SMR, 5-10, molar refractivity; Log P, 0–4, Log octanal/water partition coefficient; TPSA, <120, topological polar surface area), and then molecular docking was performed with the auxin receptor protein TIR1 (Protein Data Bank code: 2P1O) using dock module, the docking site was set as IAA/NAA-binding pocket involving residues Arg A403 and Ser 438.

### Synthetic Procedures

General procedure for the synthesis of 2-(one-benzylthio) acetic acid (Reddy et al., 2011). To a solution of thioglycolic acid (0.29 g, 3.17 mmol, 1 eqv) and 1-(bromomethyl)-3-methylbenzene (0.59 g, 3.17 mmol, 1 eqv) in ethanol (20 mL) was added dropwise in a solution of NaOH (0.38 g, 9.51 mmol, 3 eqv) in H_2_O (5 mL). The reaction was refluxed for 3 h, then ethanol was removed, after which it was poured into H_2_O and acidified by HCl (6 M) to pH 1–2. The aqueous phase was extracted with ethyl acetate (3 × 30 mL), and the combined organic phase dried over MgSO_4_ and concentrated to get compound 2 (yellow oily liquid). The crude product was used directly in the next reaction without purification. The other 2-(one-benzylthio) acetic acid were prepared in the same way above.

General procedure for the synthesis of *N*-(benzo[*d*] [1,3] dioxol-5-yl)-2-(one -benzylthio) acetamide (Sharma et al., 2004). To a solution of the crude product 2-((3-methylbenzyl) thio) acetic acid in dichloromethane (20 mL) was added dropwise in oxalyl chloride (0.48 g, 3.80 mmol, 1.2 eqv) at 0°C. The reaction was stirred for 0.5 h at 0°C, then was stirred for 1 h at room temperature, after which excessive oxalyl chloride and dichloromethane was removed to get the crude compound 3. The crude compound 3 was added dropwise to a solution of benzo[*d*] [1,3] dioxol-5-amine (0.43 g, 3.17 mol, 1 eqv) and triethylamine (0.64 g, 6.34 mol, 2 eqv) in dioxane (20 mL) at 0°C. The reaction was stirred for 0.5 h at 0°C, then was stirred for 2 h at room temperature, after it was poured into H_2_O and acidified by HCl (6 M) to pH 4-5. The aqueous phase was extracted with dichloromethane (3 × 30 mL), and the combined organic phase dried over MgSO_4_ and concentrated. The residue was purified by column chromatography to obtain compound K-16, yellow solid (0.62 g, 62%), m.p. 55.2-55.5°C. ^1^H NMR (300 MHz, DMSO) δ 8.37 (s, 1H), 7.19 (m, 2H), 7.07 (m, 3H), 6.73 (d, *J* = 1.6 Hz, 2H), 5.96 (s, 2H), 3.75 (s, 2H), 3.29 (s, 2H), 2.31 (s, 3H). ^13^C NMR (75 MHz, DMSO) δ 166.08, 147.44, 144.11, 138.29, 136.54, 131.37, 129.24, 128.41, 128.04, 125.63, 112.59, 107.62, 102.19, 100.94, 37.12, 36.05, 20.97. HRMS (ESI^–^): m/z: 338.0825 [M + Na]^+^.

The other *N*-(benzo[*d*] [1,3] dioxol-5-yl)-2-(one-benzylthio) acetamides were prepared in the same way above. The detailed characterization data (^1^H and ^13^C NMR spectra, HRMS spectra and melting points) of the prepared compounds are shown in the Supplementary Information.

### Root Phenotypic Investigation and Quantification

To explore bioactivity and the action mechanism of target compounds, the dicotyledonous model plant *A. thaliana* (Columbia-0; *DR5:GUS*; *tir1*; *yucQ*) and the monocotyledonous model plant *O. sativa* (Nihonbare) were cultured (Yang et al., 2019, 2021). *A. thaliana* seeds were sterilized in aqueous sodium hypochlorite (m/v: 1%) solution for 15 min and washed three times with sterile water, and then maintained at 4°C for 3 days under darkness. The seeds were sown onto 1/2 Murashige-Skoog (MS) culture medium sterilized in a high-pressure steam sterilization pot, containing 0.8% (m/v) agar, 1% (m/v) sucrose, and compounds with the indicated concentration. NAA was used as the reference control, and three biological replicates were performed. Subsequently, the plants were cultured on vertically oriented plates in the illumination box at 22°C for 5/7/14 days under 16 h light/8 h darkness. Primary root lengths of 7-day-old seedlings (Columbia-0; *tir1*; *yucQ*) were measured using ImageJ software after image acquisition. The root growth situation of 14-day-old seedlings was investigated using WinRHIZO root scanner. For *GUS* histochemical analysis, primary roots of 5-day-old seedlings (*DR5:GUS*) treated with 0.001 μM NAA and 0.1 μM K-10 were transferred to *GUS*-staining buffer (0.5 mM K_4_Fe[CN]_6_, 0.5 mM K_3_Fe[CN]_6_, 100 mM sodium phosphate, pH 7.0, 10 mM EDTA and 0.1% [v/v] Triton X-100) containing 1 mM X-gluc and incubated at 37°C for 15 h. The staining areas and numbers of root primordium were measured using ImageJ software after image acquisition.

*Oryza sativa* seeds were sterilized in aqueous sodium hypochlorite (m/v: 1%) solution for 30 min and washed three times with sterile water, and then maintained in water at 30°C for 24 h under darkness. In order to explore preliminary bioactivity of target compounds and the dose curve of K-10 and NAA on *O. sativa*, the treated seeds sown on the medium containing 1% agar and the compounds with the indicated concentrations after germinating for 2 days. NAA was used as the reference control, and three biological replicates were performed. Subsequently, the cultivating boxes were placed for 5 days in the incubator at 30°C under 16 h light/8 h darkness. The primary root lengths and root numbers of 7-day-old seedlings were investigated. To further explore the symptomology of *O. sativa* treated with K-10 in different periods, the treated seeds sown on the medium containing 1% agar and then were cultured for 5 days in the incubator at 30°C under 16 h light/8 h darkness. Subsequently, the seedlings were transferred to the new mediums containing 1% agar and the compounds with the indicated concentrations, the cultivating boxes were placed for 5 days in the incubator at 30°C under 16 h light/8 h darkness. NAA was used as the reference control, and three biological replicates were performed. The root growth situation of 10-day-old seedlings was investigated using WinRHIZO root scanner.

For root sampling of cellular morphology, transcriptomics and metabolomics analysis, the treated seeds sown in wet quartz sand for 5 days at 30°C under darkness and then the seedlings were transferred to the culture box (30 cm × 20 cm) filled with 5 L nutrient solution (Supplementary Table 1). Twenty-four seedlings were placed in each box, and three biological replicates were performed. The seedlings were cultured in the greenhouse at 25–30°C under 14 h light (400 μmol/m2/s)/10 h darkness, and the nutrient solution was replaced every 5 days. Additionally, emulsions of K-10 and NAA were prepared by dissolving them in DMSO (0.1%) adding Tween-80 (0.5%) and dispersing in water. A mixture of the same amount of water, DMSO and Tween-80 was used as the control check. Subsequently, the emulsion was added to the culture medium when the nutrient solution was replaced for the fourth time. Root samples were taken after 6, 12, and 24 h post treatment and stored in liquid nitrogen. Then 27 samples were sent to Mega Biotech for transcriptome sequencing. Root samples were taken after 24 h post treatment, and the endogenous IAA concentrations of root were measured using a high-performance liquid chromatography-mass spectrometry. Root samples were taken after 5 days post treatment and stored in FFA fixative solution. The root growth situation of 25-day-old seedlings was investigated using WinRHIZO root scanner.

### *Oryza sativa* Root Structure and Endogenous IAA Concentrations Analysis

The embryonic adventitious root tissues of *O. sativa* 1.5 cm from the root tip were embedded in paraffin and cut into 100 μm slices with vibrating microtome (Coudert et al., 2010). The tissue slices were maintained in 0.01% toluidine blue o for 1 min and then rinsed with sterile water. The tissue slices were placed on glass slides and covered with a cover glassed to be temporary sections. The root sections were observed and photographed with scanning microscope, the total root area and vascular cylinder area were investigated by ImageJ software. Endogenous IAA was extracted overnight in 50% acetonitrile from the root samples. The extracting solutions were purified by MAX solid phase extraction column (Waters, United States). An ACQUITY UPLC System (Waters, United States) combined with a Q Exactive mass spectrometer (Thermo Fisher Scientific, United States) was used for quantification. Samples were injected on a HSS T3 liquid chromatography column (50 mm × 2.1 mm, 1.8 μm; Waters, United States): the injection volume was 2 μL; the column temperature was 40°C; the mobile phase A was 1:999 acetic acid:acetonitrile; the mobile phase B was 1:999 acetic acid:water. After 2 min equilibration at 10:90 A:B, samples were eluted from the column by changing solvent composition to 90:10 A:B in a 2 min linear gradient, using a constant flow-rate of 0.3 mL min^–1^. ESI (+)-MRM mode was used for quantification for IAA (190 > 130). Chromatograms obtained were processed using QuanLynx v4.1 (Waters, United States). Concentrations were calculated using the standard curve. In the experiment, the external standard method was used for quantitative analysis. The fitting curve was prepared by the standard substances of different concentrations. The information of the standard curve was as follows: Y = 5.294 e^4^ X, R^2^ = 0.9978.

### Transcriptome Analysis

Total RNA samples were sequenced by Majorbio using the Illumina Novaseq 6000 sequencing platform, with 10 M reads per sample with average length >50 bp. All reads were mapped to the *O. sativa* (IRGSP-1.0) by TopHat2. Mapped reads were assembled and spliced to analyze gene expression values by Cufflinks. The differential expression genes (DEGs) analysis was performed using DESeq2, | log_2_FC| ≥ 0.848 and FDR < 0.1. Fold change (FC) was computed with average transcript levels compared to DMSO control values, which was in turn log_2_-transformed and computed for Spearman correlation coefficients between samples. Gene ontology analysis was performed using the free online platform of Majorbio Cloud Platform. The transcriptomic data are available on National Center for Biotechnology Information.^4^

### Molecular Docking Simulation

Molecules were drawn and minimized with SYBYL 7.2 software. The receptor protein TIR1 (Protein Data Bank code: 2P1O) was prepared by the process of deleting water, adding hydrogen, adding Gasteiger charges, merging non-polar hydrogen and so on by AutodockTools software. AutoGrid was run to identify the pocket with the parameters: center grid box (–3.139, 8.278, –3.111), 60 × 60 × 60, spacing 0.375 angstrom, where was IAA/NAA-binding pocket involving residues Arg A403 and Ser 438. Molecular docking was operated after setting rigid filename and choosing Genetic Algorithm with number of GA Runs: 200. The docking results were visualized by Molecular Operate Environment software.

### Statistical Analysis

Microsoft Excel 2019, Origin Pro 9.0 (Originlab Corporation, United States) and SPSS 20 (IBM, United States) were used as the statistical software program. Student’s t test was used as the statistical test.

## Conclusion

In summary, a series of *N*-(benzo[*d*][1,3]dioxol-5-yl)-2-(one-benzylthio) acetamide compounds was designed based on the lead compound HTS05309 screened from the MayBridge Screening Collection. Twenty-two compounds were synthesized and tested in *Arabidopsis* and *Oryza sativa*, K-10 displayed an excellent root growth-promoting activity far exceeding that of NAA, and the study on *Arabidopsis* mutant (*yucQ*), auxin reporter (*DR5:GUS*) and endogenous IAA concentration revealed that K-10 possesses the auxin-like physiological functions and significantly enhanced auxin response reporter’s (*DR5:GUS*) transcriptional activity. Furthermore, the mutant (*tir1*) was insensitive to K-10 application, indicating that K-10 was an auxin receptor agonist. Consistently, transcriptome analysis showed that K-10 induced a common transcriptional response with auxin and exhibited a unique regulation mechanism that down-regulated the expression of root growth-inhibiting genes. Finally, molecular docking analysis revealed that K-10 had a stronger binding ability with TIR1 than NAA, resulting in enhanced auxin signaling. These results suggest that K-10 might be a potential root growth promoter acting as an auxin receptor agonist, and it provides a molecular basis for future regulator designs and may be part of an efficient management approach for regulating root phenotypes in agricultural production.

## Data Availability Statement

The original contributions presented in the study are included in the article/Supplementary Material, further inquiries can be directed to the corresponding authors.

## Author Contributions

ZY, KS, and WT conceived and designed the experiments. ZY performed computer-aid, the synthetic experiments, and wrote the manuscript. ZY, JX, JY, and ZW performed the bioassays. ZY, FY, KS, and LD analyzed the data. KS, WT, BW, LD, and ZL revised the manuscript. All authors contributed to the article and approved the submitted version.

## Conflict of Interest

The authors declare that the research was conducted in the absence of any commercial or financial relationships that could be construed as a potential conflict of interest.

## Publisher’s Note

All claims expressed in this article are solely those of the authors and do not necessarily represent those of their affiliated organizations, or those of the publisher, the editors and the reviewers. Any product that may be evaluated in this article, or claim that may be made by its manufacturer, is not guaranteed or endorsed by the publisher.

## Footnotes

1
https://imagej.nih.gov/ij/index.html

2
www.majorbio.com

3
https://www.thermofisher.cn

4
https://dataview.ncbi.nlm.nih.gov/object/PRJNA706670?reviewer=8obqp31phl6thtdn8sebqegkhd
