# Supplementary material for: Design, Synthesis, and Action Mechanism of 1,3-Benzodioxole Derivatives as Potent Auxin Receptor Agonists and Root Growth Promoters
Source: Front Plant Sci. 2022 Jun 10;13:902902. doi: 10.3389/fpls.2022.902902 (PMC9226723; doi:10.3389/fpls.2022.902902)
Supplement: Supplementary file 1 [file Data_Sheet_1.docx]

**Design, Synthesis, and Action Mechanism of 1,3-benzodioxole Derivatives as Potent Auxin Receptor Agonists and Root Growth Promoters**

Zhikun Yang^1,3^ Jiahui Xu^1,2^ Lin Du^1^ Jiaming Yin^1^ Zhao Wang^1^ Fei Yi^1^ Liusheng Duan^1^ Zhaohu Li^1^ Baomin Wang^1^ Kai Shu^2,*^ and Weiming Tan^1,*^

1. College of Agronomy and Biotechnology, China Agricultural University, Beijing 100193, China.

2. School of Ecology and Environment, Northwestern Polytechnical University, Xi'an 710129, China.

3. College of Pharmaceutical Science & Green Pharmaceutical Collaborative Innovation Center of Yangtze River Del-ta Region, Zhejiang University of Technology, Hangzhou 310014, China.

***Corresponding author**

Dr. Weiming Tan, E-mail: [tanwm@cau.edu.cn](mailto:tanwm@cau.edu.cn)

Dr. Kai Shu, E-mail: kshu@sicau.edu.cn

**SUPPLEMENTARY INFORMATION**

**Supplementary Methods**

The detailed characterization data of target compounds

**Supplementary Results**

Figure. S1. Pharmacophore model used for virtual screening

Figure. S2. Heat map of correlation between 18 samples

Figure. S3. GO enrichment analysis of DEGs from root treated with NAA (A, B, C) and K-10 (D, E, F) at 6, 12 and 24 h

Figure. S4. Heat map of auxin-related genes (*GH3/YUC, LAX, Aux/IAA, SAUR, TIR1*) expression levels in rice roots treated with NAA or K-10 at 6, 12 and 24 h

Figure. S5. Cluster of 200 docked conformations for NAA (A) and K-10 (B)

Figure. S6. The ^1^H NMR and ^13^C NMR spectrum of target compounds

Table S1. The statistical chart of rice hydroponic culture solution

**Supplementary Methods**

The detailed characterization data of target compounds

Compound K-1, white solid (0.85 g, 85%), m.p. 87.2-87.5 ℃. ^1^H NMR (300 MHz, CDCl_3_) δ 8.45 (s, 1H), 7.65 (d, *J* = 7.8 Hz, 1H), 7.53 – 7.47 (m, 2H), 7.40 – 7.31 (m, 1H), 7.18 (s, 1H), 6.75 (s, 2H), 5.96 (s, 2H), 3.98 (s, 2H), 3.37 (s, 2H). ^13^C NMR (75 MHz, CDCl_3_) δ 165.73, 147.47, 144.20, 135.01, 131.84, 131.49, 131.23, 128.15 (q, *J* = 60.3, 30.3 Hz), 127.45, 126.28 (q, *J* = 5.6 Hz), 125.78, 112.64, 107.68, 102.20, 100.95, 36.58, 33.84. HRMS (ESI^-^): m/z: 370.0723 [M+H]^+^.

Compound K-2, brown solid (0.89 g, 89%), m.p. 78.6-79.3 ℃. ^1^H NMR (300 MHz, CDCl_3_) δ 8.29 (s, 1H), 7.61 – 7.31 (m, 5H), 7.16 (t, *J* = 1.1 Hz, 1H), 6.72 (d, *J* = 1.1 Hz, 2H), 5.94 (s, 2H), 3.83 (s, 2H), 3.25 (s, 2H). ^13^C NMR (75 MHz, CDCl_3_) δ 165.91, 147.50, 144.24, 137.74, 132.01, 131.17, 130.71, 129.00, 128.89, 125.26 (q, *J* = 7.6, 3.9 Hz), 124.06 (q, *J* = 3.8 Hz), 112.60, 107.68, 102.16, 100.99, 36.43, 35.85. HRMS (ESI^-^): m/z: 370.0706 [M+H]^+^.

Compound K-3, white solid (0.82 g, 82%), m.p. 97.6-98.7 ℃. ^1^H NMR (300 MHz, CDCl_3_) δ 8.23 (s, 1H), 7.56 (d, *J* = 8.2 Hz, 2H), 7.42 (d, *J* = 8.1 Hz, 2H), 7.14 (d, *J* = 1.9 Hz, 1H), 6.70 (m, 2H), 5.96 (s, 2H), 3.83 (s, 2H), 3.26 (s, 2H). ^13^C NMR (75 MHz, CDCl_3_) δ 165.81, 147.53, 144.28, 140.85, 131.11, 129.48 (q, *J* = 52.1, 19.6 Hz), 128.93, 125.42 (q, *J* = 3.8 Hz), 121.81, 112.53, 107.67, 102.10, 100.99, 36.44, 35.89. HRMS (ESI^-^): m/z: 370.0723 [M+H]^+^.

Compound K-4, white solid (0.71 g, 71%), m.p. 54.0-55.1 ℃. ^1^H NMR (300 MHz, CDCl_3_) δ 8.52 (s, 1H), 7.34 (m, 1H), 7.23 (m, 3.2 Hz, 1H), 7.17 (s, 1H), 7.12 – 6.99 (m, 2H), 6.75 (s, 2H), 5.96 (s, 2H), 3.83 (s, 2H), 3.34 (s, 2H). ^13^C NMR (75 MHz, CDCl_3_) δ 165.92, 160.51 (d, *J* = 247.2 Hz), 147.44, 144.16, 131.28, 130.70 (d, *J* = 3.6 Hz), 129.23 (d, *J* = 8.2 Hz), 124.09 (d, *J* = 3.7 Hz), 123.81 (d, *J* = 14.5 Hz), 115.41 (d, *J* = 21.4 Hz), 112.70, 107.65, 102.24, 100.94, 36.20, 30.35 (d, *J* = 2.9 Hz). HRMS (ESI^-^): m/z: 320.0755 [M+H]^+^.

Compound K-5, yellow solid (0.83 g, 83%), m.p. 55.5-56.0 ℃. ^1^H NMR (300 MHz, CDCl_3_) δ 8.32 (s, 1H), 7.30 – 7.21 (m, 1H), 7.17 (s, 1H), 7.08 – 7.00 (m, 2H), 6.96 – 6.91 (m, 1H), 6.74 (d, *J* = 1.0 Hz, 2H), 5.95 (s, 2H), 3.76 (s, 2H), 3.25 (s, 2H). ^13^C NMR (75 MHz, CDCl_3_) δ 165.88, 162.63 (d, *J* = 247.0 Hz), 147.50, 144.20, 139.09 (d, *J* = 7.4 Hz), 131.26, 129.96 (d, *J* = 8.3 Hz), 124.32 (d, *J* = 2.9 Hz), 115.44 (d, *J* = 21.7 Hz), 114.28 (d, *J* = 21.0 Hz), 112.60, 107.69, 102.19, 100.98, 36.46 (d, *J* = 1.4 Hz), 35.81. HRMS (ESI^-^): m/z: 320.0743 [M+H]^+^.

Compound K-6, yellow solid (0.66 g, 66%), m.p. 70.0-70.6 ℃. ^1^H NMR (300 MHz, CDCl_3_) δ 8.40 (s, 1H), 7.32 – 7.27 (m, 2H), 7.19 (t, *J* = 1.2 Hz, 1H), 7.01 (t, *J* = 8.7 Hz, 2H), 6.77 (d, *J* = 1.2 Hz, 2H), 5.98 (s, 2H), 3.78 (s, 2H), 3.26 (s, 2H). ^13^C NMR (75 MHz, CDCl_3_) δ 166.11, 161.81 (d, *J* = 246.6 Hz), 147.52, 144.21, 132.32 (d, *J* = 3.2 Hz), 131.27, 130.23 (d, *J* = 8.2 Hz), 115.36 (d, *J* = 21.6 Hz), 112.64, 107.69, 102.21, 100.99, 36.12, 35.72. HRMS (ESI^-^): m/z: 320.0755 [M+H]^+^.

Compound K-7, brown solid (0.72 g, 72%), m.p. 65.0-65.7 ℃. ^1^H NMR (300 MHz, DMSO) δ 8.57 (s, 1H), 7.35 – 7.26 (m, 2H), 7.20 – 7.10 (m, 3H), 6.76 – 6.65 (m, 2H), 5.91 (s, 2H), 3.88 (s, 2H), 3.28 (s, 2H). ^13^C NMR (75 MHz, CDCl_3_) δ 166.12, 147.39, 144.06, 134.23, 133.59, 131.37, 130.77, 129.67, 128.75, 126.78, 112.66, 107.63, 102.17, 100.94, 36.07, 34.83. HRMS (ESI^-^): m/z: 336.0447 [M+H]^+^.

Compound K-8, yellow solid (0.80 g, 80%), m.p. 64.8-65.1 ℃. ^1^H NMR (300 MHz, DMSO) δ 8.33 (s, 1H), 7.31 (s, 1H), 7.24 – 7.12 (m, 4H), 6.73 (d, *J* = 1.1 Hz, 2H), 5.94 (s, 2H), 3.74 (s, 2H), 3.24 (s, 2H). ^13^C NMR (75 MHz, CDCl_3_) δ 165.97, 147.49, 144.21, 138.72, 134.35, 131.24, 129.66, 128.61, 127.44, 126.82, 112.67, 107.68, 102.22, 100.98, 36.39, 35.84. HRMS (ESI^-^): m/z: 336.0443 [M+H]^+^.

Compound K-9, yellow solid (0.68 g, 68%), m.p. 70.9-71.5 ℃. ^1^H NMR (300 MHz, CDCl_3_) δ 8.46 (s, 1H), 7.55 – 7.50 (m, 1H), 7.33 – 7.27 (m, 1H), 7.25 – 7.05 (m, 3H), 6.73 (d, *J* = 1.2 Hz, 2H), 5.95 (s, 2H), 3.91 (s, 2H), 3.34 (s, 2H). ^13^C NMR (75 MHz, CDCl_3_) δ 165.78, 147.42, 144.08, 135.76, 133.05, 131.31, 130.77, 128.99, 127.46, 124.00, 112.53, 107.64, 102.08, 100.94, 37.70, 36.21. HRMS (ESI^-^): m/z: 379.9952 [M+H]^+^.

Compound K-10, brown solid (0.73 g, 73%), m.p. 81.3-82.0 ℃. ^1^H NMR (300 MHz, CDCl_3_) δ 8.42 (s, 1H), 7.46 (t, *J* = 1.6 Hz, 1H), 7.35 – 7.29 (m, 1H), 7.22 – 7.11 (m, 3H), 6.74 – 6.70 (m, 2H), 5.91 (s, 2H), 3.72 (s, 2H), 3.21 (s, 2H). ^13^C NMR (75 MHz, CDCl_3_) δ 166.33, 147.44, 144.19, 139.13, 131.52, 131.26, 130.29, 129.92, 127.33, 122.48, 112.85, 107.68, 102.34, 100.99, 36.26, 35.77. HRMS (ESI^-^): m/z: 379.9940 [M+H]^+^.

Compound K-11, brown solid (0.45 g, 45%), m.p. 91.2-92.0 ℃. ^1^H NMR (300 MHz, CDCl_3_) δ 8.30 (s, 1H), 7.48 – 7.40 (m, 2H), 7.23 – 7.15 (m, 3H), 6.81 – 6.70 (m, 2H), 5.98 (s, 2H), 3.75 (s, 2H), 3.26 (s, 2H). ^13^C NMR (75 MHz, CDCl_3_) δ 165.92, 147.52, 144.21, 135.70, 131.60, 131.22, 130.27, 121.22, 112.63, 107.71, 102.19, 100.99, 36.32, 35.82. HRMS (ESI^-^): m/z: 379.9949 [M+H]^+^.

Compound K-12, yellow solid (0.37 g, 37%), m.p. 81.2-82.1 ℃. ^1^H NMR (300 MHz, CDCl_3_) δ 8.46 (s, 1H), 7.82 (d, *J* = 8.2 Hz, 1H), 7.34 – 7.28 (m, 2H), 7.18 (s, 1H), 6.97 – 6.85 (m, 1H), 6.74 (s, 2H), 5.96 (s, 2H), 3.91 (s, 2H), 3.35 (s, 2H). ^13^C NMR (75 MHz, CDCl_3_) δ 165.77, 147.42, 144.09, 139.77, 138.83, 131.31, 130.03, 129.01, 128.32, 112.61, 107.63, 102.14, 100.93, 99.92, 42.52, 36.21. HRMS (ESI^-^): m/z: 427.9815 [M+H]^+^.

Compound K-13, yellow solid (0.52 g, 52%), m.p. 98.4-99.4 ℃. ^1^H NMR (300 MHz, CDCl_3_) δ 8.27 (s, 1H), 7.68 (t, *J* = 1.7 Hz, 1H), 7.55 (d, *J* = 7.9 Hz, 1H), 7.26 – 7.15 (m, 2H), 7.02 (t, *J* = 7.8 Hz, 1H), 6.73 (d, *J* = 1.9 Hz, 2H), 5.95 (s, 2H), 3.70 (s, 2H), 3.26 (s, 2H). ^13^C NMR (75 MHz, CDCl_3_) δ 165.78, 147.50, 144.20, 139.02, 137.39, 136.32, 131.21, 130.07, 127.88, 112.62, 107.71, 102.19, 100.98, 94.39, 36.30, 35.98. HRMS (ESI^-^): m/z: 427.9807 [M+H]^+^.

Compound K-14, yellow solid (0.46 g, 46%), m.p. 110.2-111.0 ℃. 1H NMR (300 MHz, MeOD) δ 8.33 (s, 1H), 7.63 (d, J = 8.3 Hz, 2H), 7.16 (d, J = 1.9 Hz, 1H), 7.06 (d, J = 8.3 Hz, 2H), 6.80 – 6.68 (m, 2H), 5.98 (s, 2H), 3.73 (s, 2H), 3.25 (s, 2H). 13C NMR (75 MHz, CDCl3) δ 165.98, 147.50, 144.20, 137.57, 136.44, 131.21, 130.50, 112.66, 107.74, 102.20, 101.00, 92.71, 36.47, 35.86. HRMS (ESI^-^): m/z: 427.9810 [M+H]^+^.

Compound K-15, white solid (0.73 g, 73%), m.p. 62.7-63.4 ℃. ^1^H NMR (300 MHz, CDCl_3_) δ 8.41 (s, 1H), 7.38 – 7.32 (m, 4H), 7.32 – 7.27 (m, 1H), 7.18 (t, *J* = 1.2 Hz, 1H), 6.76 (d, *J* = 1.1 Hz, 2H), 5.97 (s, 2H), 3.81 (s, 2H), 3.29 (s, 2H). ^13^C NMR (75 MHz, CDCl_3_) δ 166.16, 147.45, 144.15, 136.62, 131.33, 128.65 (t, *J* = 12.6 Hz), 128.23, 127.24, 112.72, 107.65, 102.29, 100.95, 37.05, 35.89. HRMS (ESI^-^): m/z: 302.0849 [M+H]^+^.

Compound K-17, red solid (0.64 g, 64%), m.p. 53.6-54.5 ℃. ^1^H NMR (300 MHz, DMSO) δ 8.33 (s, 1H), 7.21 (m, 2H), 6.87 (m, 2H), 6.80 – 6.71 (m, 3H), 5.96 (s, 2H), 3.78 (s, 3H), 3.76 (s, 2H), 3.30 (s, 2H). ^13^C NMR (75 MHz, DMSO) δ 166.01, 159.63, 147.43, 144.12, 138.17, 131.34, 129.54, 120.89, 114.14, 112.78, 112.61, 107.62, 102.20, 100.94, 54.87, 37.17, 36.06. HRMS (ESI^-^): m/z: 332.0955 [M+H]^+^.

Compound K-18, white solid (0.78 g, 78%), m.p. 95.3-95.9 ℃. ^1^H NMR (300 MHz, CDCl_3_) δ 8.39 (s, 1H), 7.33 (m, 1H), 7.24 – 7.08 (m, 3H), 6.79 – 6.68 (m, 2H), 5.97 (s, 2H), 3.93 (s, 2H), 3.33 (s, 2H). ^13^C NMR (75 MHz, CDCl_3_) δ 165.62, 147.47, 144.15, 136.41, 133.62, 131.89, 131.19, 129.58, 128.78, 127.05, 112.39, 107.68, 101.93, 100.95, 36.27, 35.91. HRMS (ESI^-^): m/z: 370.0070 [M+H]^+^.

Compound K-19, brown solid (0.82 g, 82%), m.p. 122.3-123.2 ℃. ^1^H NMR (300 MHz, CDCl_3_) δ 8.41 (s, 1H), 7.35 (d, *J* = 2.4 Hz, 1H), 7.28 (d, *J* = 5.1 Hz, 1H), 7.22 – 7.11 (m, 2H), 6.75 (s, 2H), 5.96 (s, 2H), 3.87 (s, 2H), 3.35 (s, 2H). ^13^C NMR (75 MHz, CDCl_3_) δ 165.68, 147.49, 144.19, 136.02, 132.63, 131.86, 131.22, 130.66, 130.43, 128.74, 112.54, 107.68, 102.10, 100.97, 36.24, 34.49. HRMS (ESI^-^): m/z: 370.0071 [M+H]^+^.

Compound K-20, yellow solid (0.90 g, 90%), m.p. 125.1-126.4 ℃. ^1^H NMR (300 MHz, CDCl_3_) δ 8.65 (s, 1H), 7.37 – 7.26 (m, 3H), 7.21 – 7.06 (m, 2H), 6.76 – 6.72 (m, 2H), 5.95 (s, 2H), 4.15 (s, 2H), 3.46 (s, 2H). ^13^C NMR (75 MHz, CDCl_3_) δ 165.53, 147.44, 144.06, 135.20, 132.85, 131.33, 128.90, 128.30, 112.40, 107.65, 101.97, 100.93, 36.69, 32.40. HRMS (ESI^-^): m/z: 370.0066 [M+H]^+^.

Compound K-21, yellow solid (0.54 g, 54%), m.p. 118.1-118.8 ℃. ^1^H NMR (300 MHz, DMSO) δ 8.18 (s, 1H), 7.39 (m, 2H), 7.20 – 7.09 (m, 2H), 6.74 (m, 2H), 5.97 (s, 2H), 3.73 (s, 2H), 3.26 (s, 2H). ^13^C NMR (75 MHz, CDCl_3_) δ 165.76, 147.54, 144.26, 136.97, 132.57, 131.41, 131.14, 130.40, 130.32, 127.95, 112.51, 107.72, 102.07, 101.00, 35.85, 35.83. HRMS (ESI^-^): m/z: 370.0068 [M+H]^+^.

Compound K-22, yellow solid (0.69 g, 69%), m.p. 131.2-132.5 ℃. ^1^H NMR (300 MHz, CDCl_3_) δ 8.12 (s, 1H), 7.51 (t, *J* = 1.7 Hz, 1H), 7.40 (d, *J* = 1.7 Hz, 2H), 7.21 (t, *J* = 1.3 Hz, 1H), 6.75 (d, *J* = 1.2 Hz, 2H), 5.97 (s, 2H), 3.71 (s, 2H), 3.27 (s, 2H). ^13^C NMR (75 MHz, CDCl_3_) δ 165.59, 147.54, 144.27, 140.67, 132.90, 131.15, 130.32, 122.89, 112.55, 107.72, 102.14, 101.01, 35.92, 35.85. HRMS (ESI^-^): m/z: 457.9058 [M+H]^+^.

**Supplementary Results**


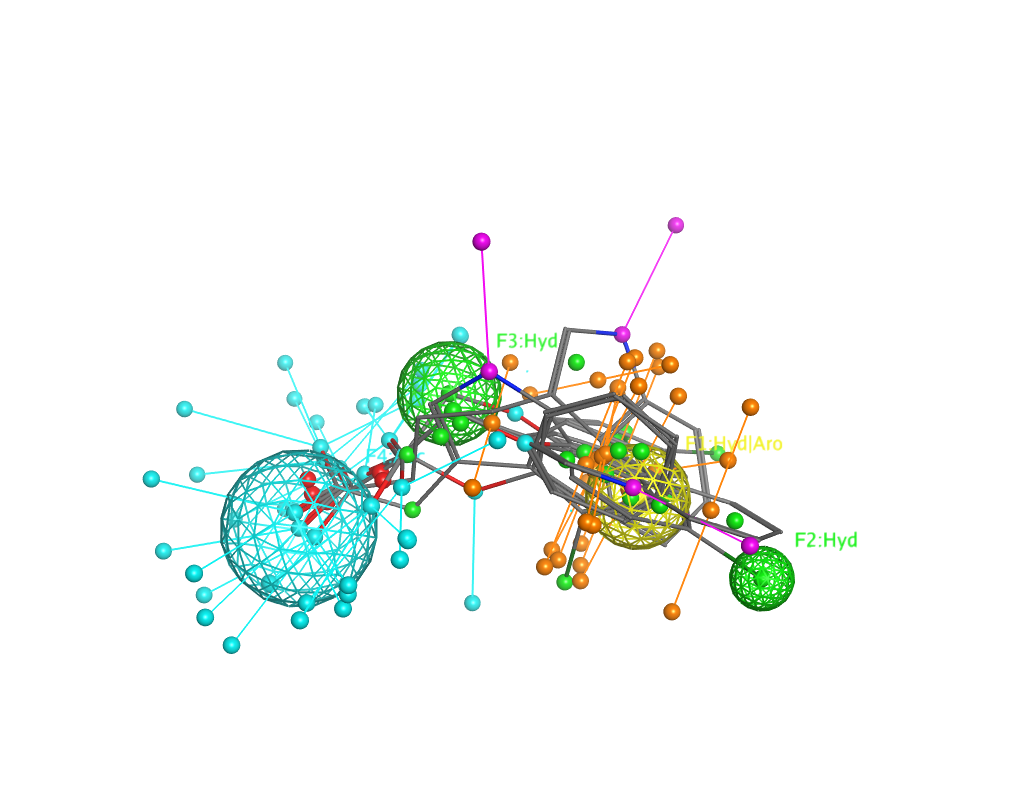


Figure. S1. Pharmacophore model used for virtual screening.


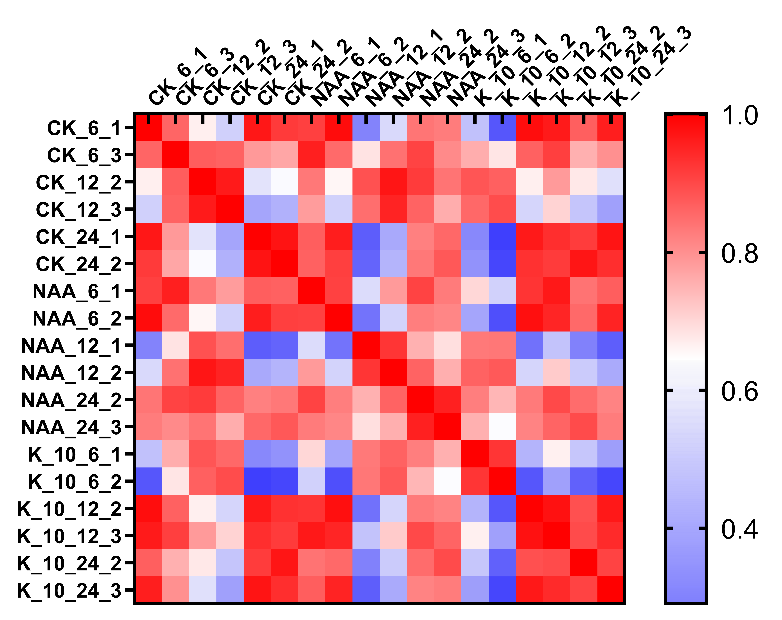


Figure. S2. Heat map of correlation between 18 samples.

(A)
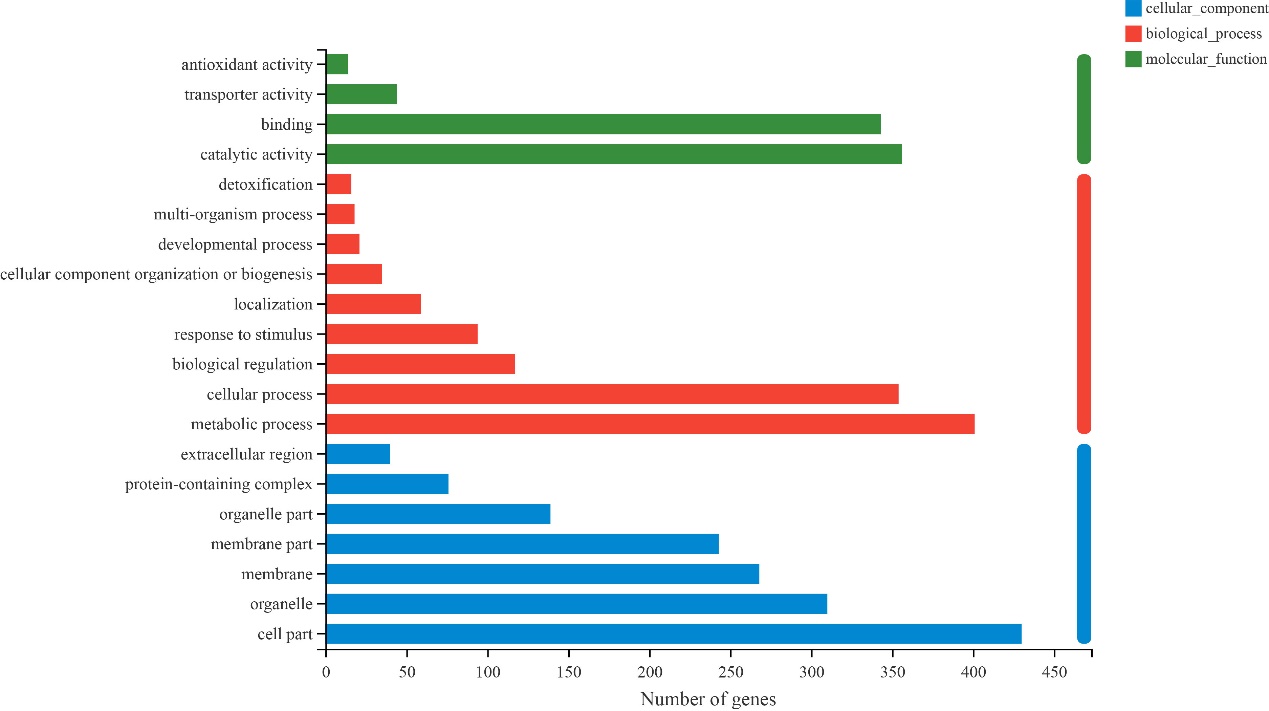


(B)
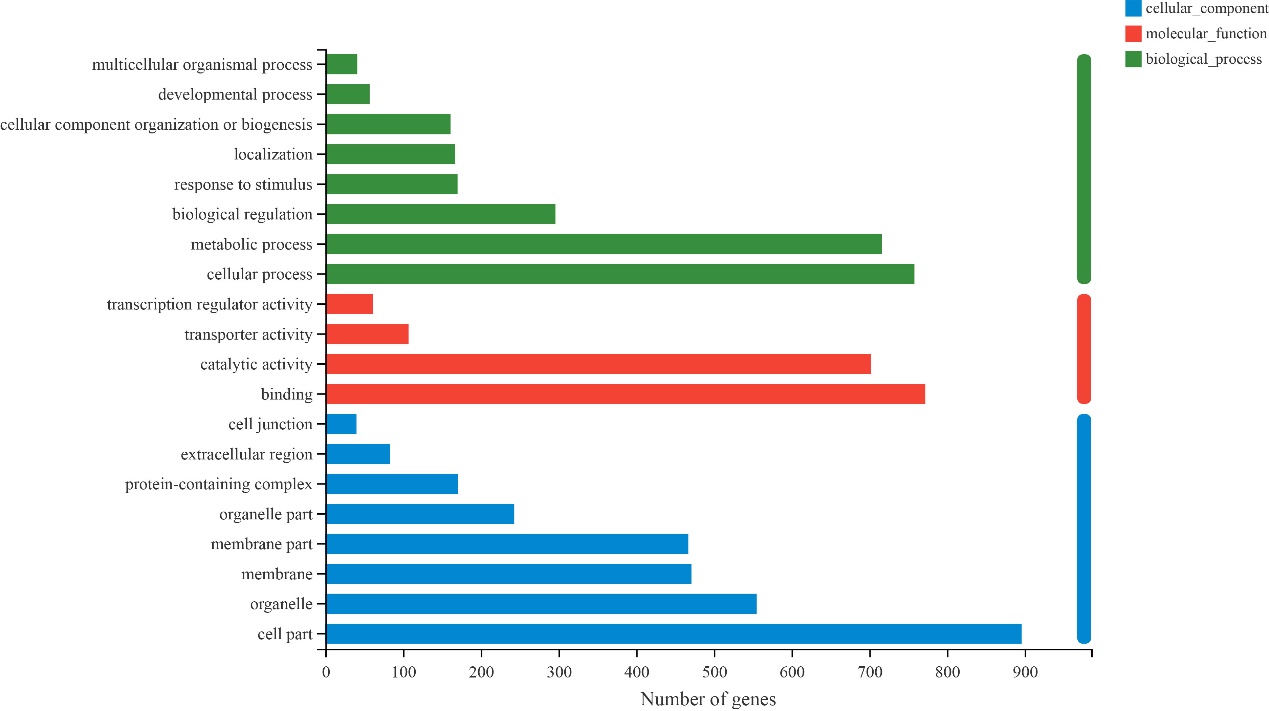


(C)
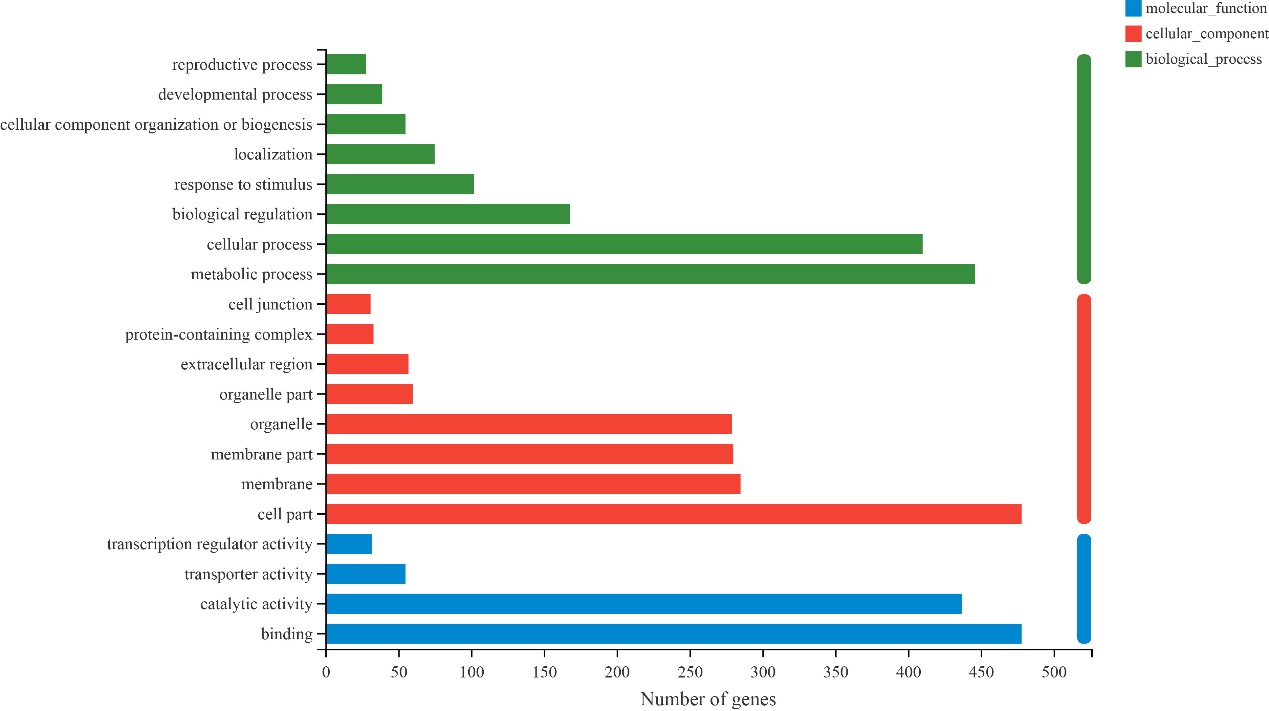


(D)
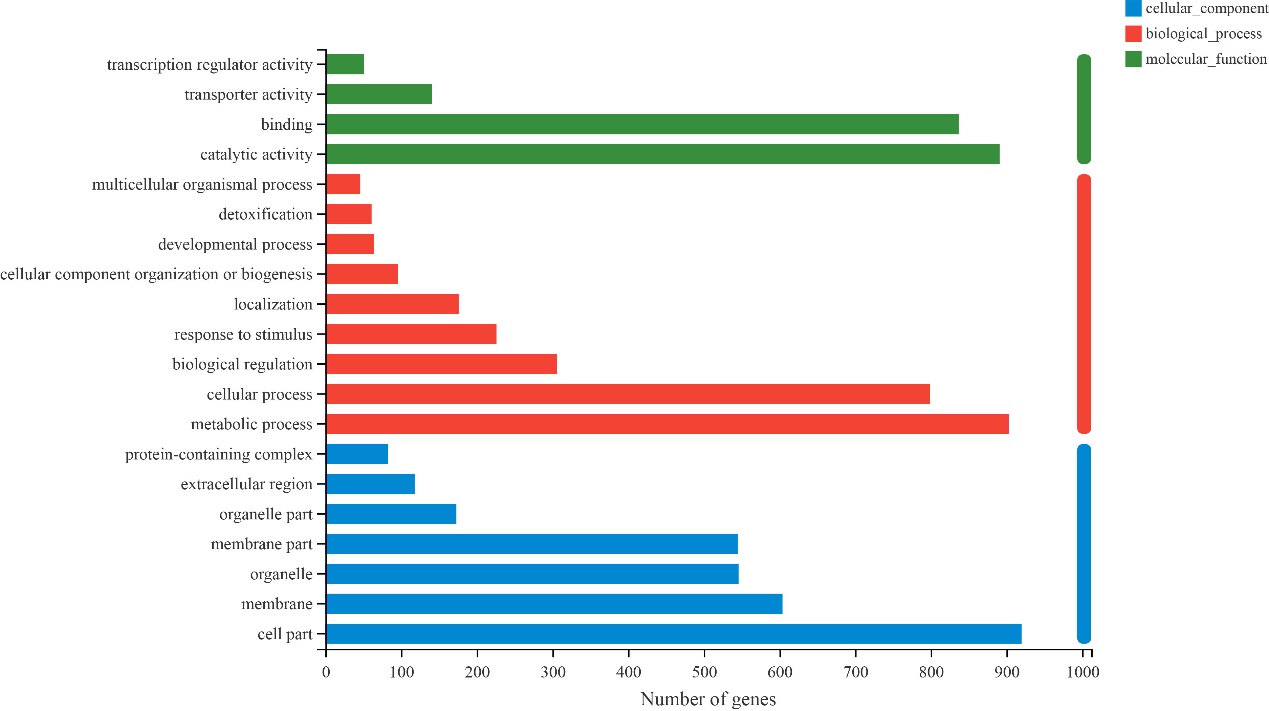


(E)
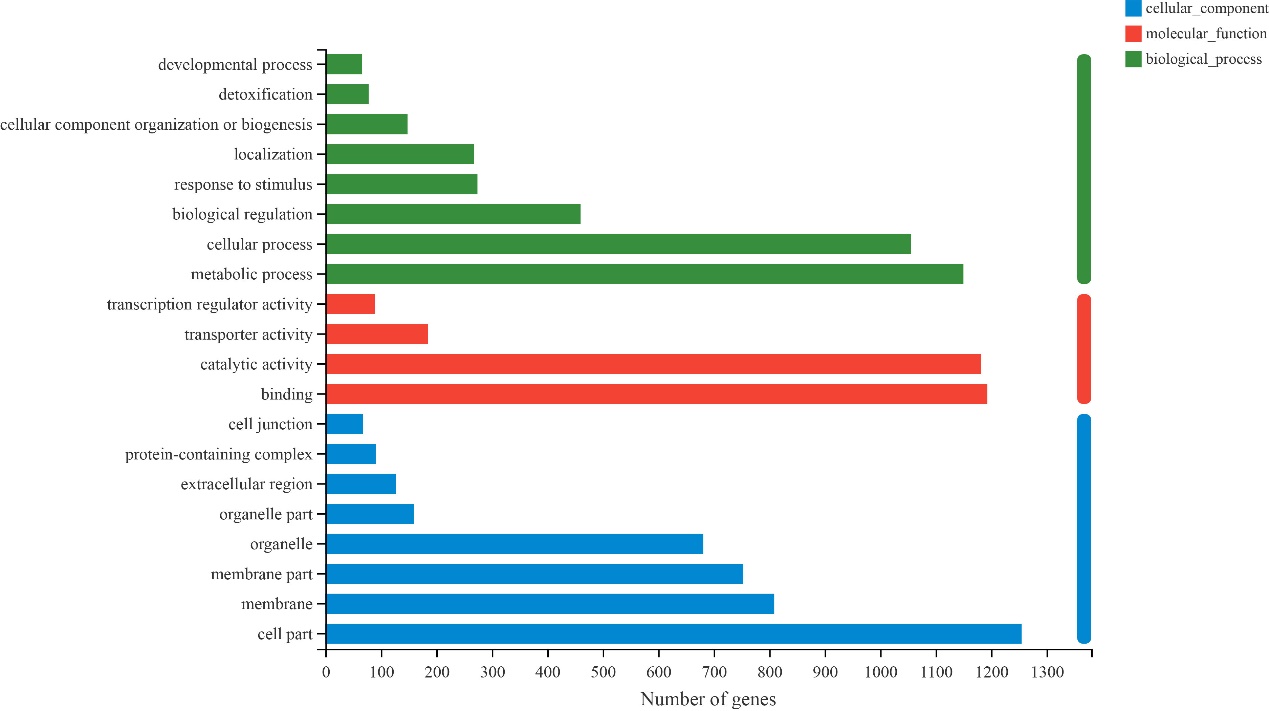


(F)
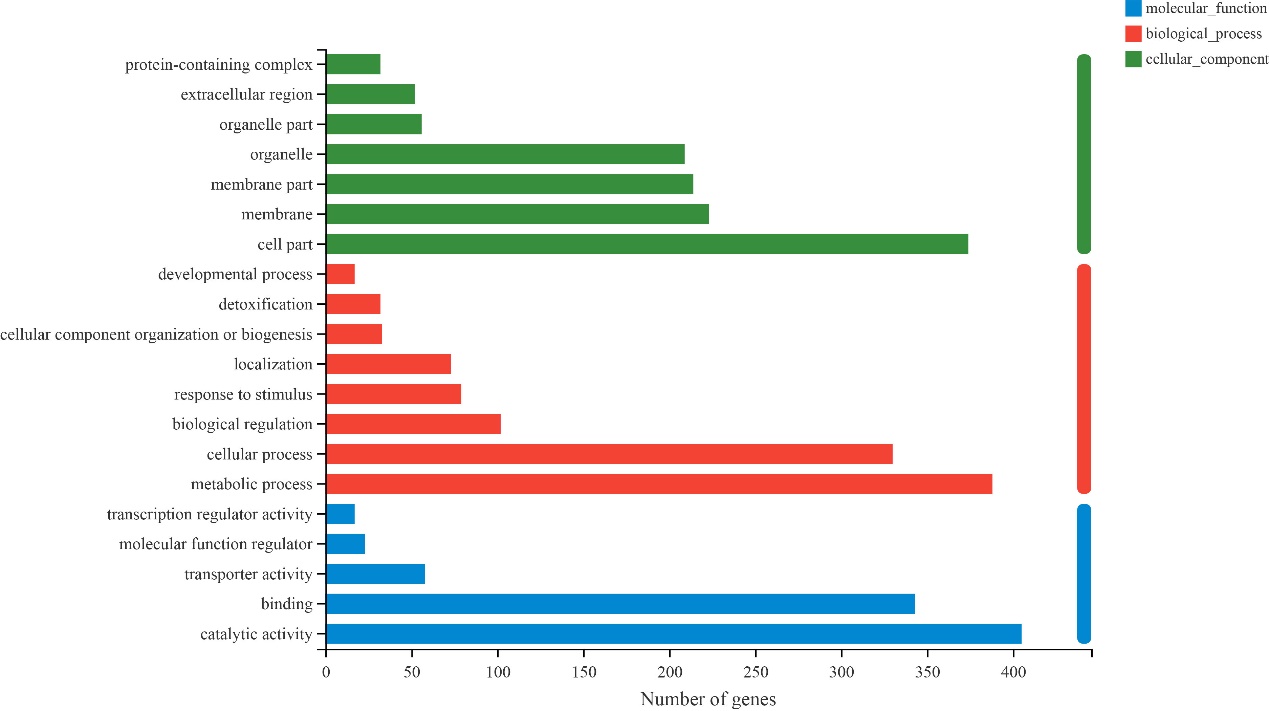


Figure. S3. GO enrichment analysis of DEGs from root treated with NAA (A, B, C) and K-10 (D, E, F) at 6, 12 and 24 h.


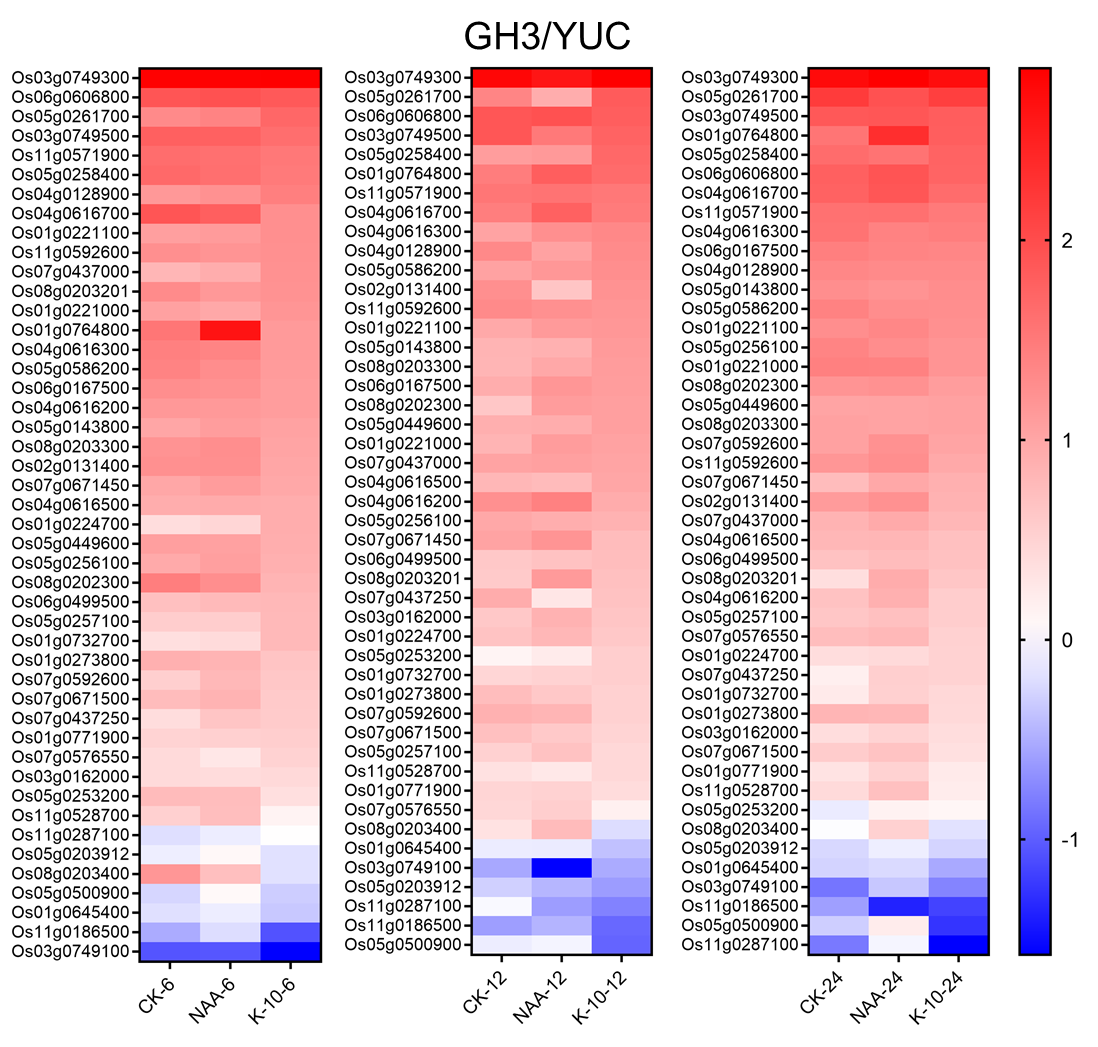


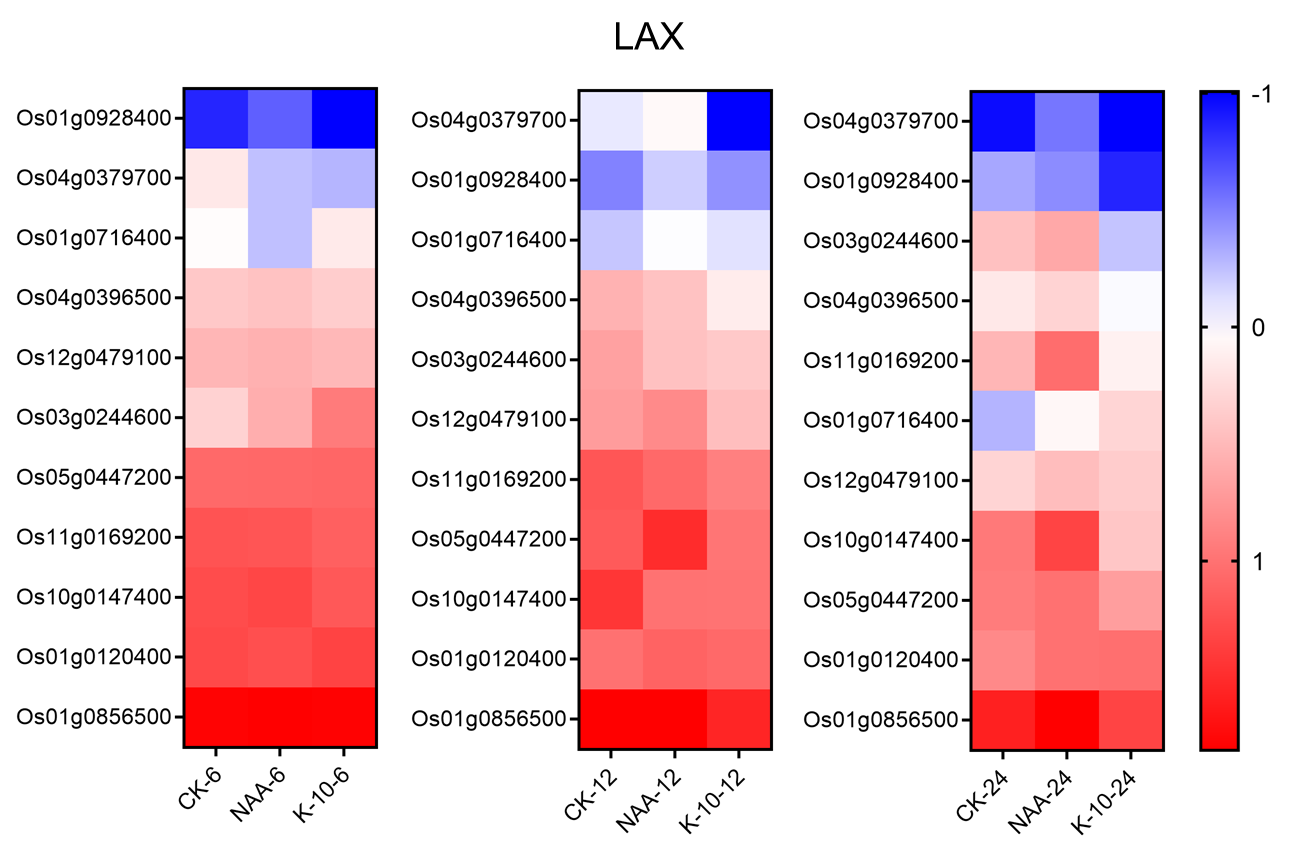


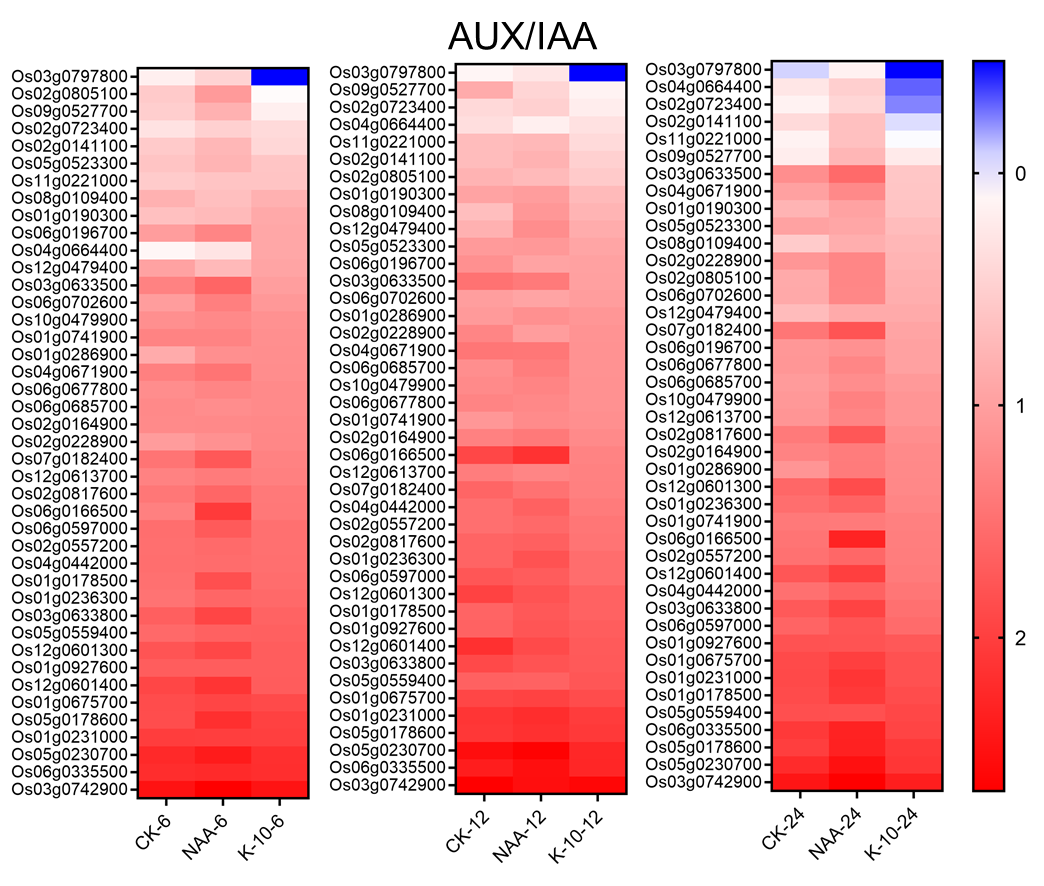


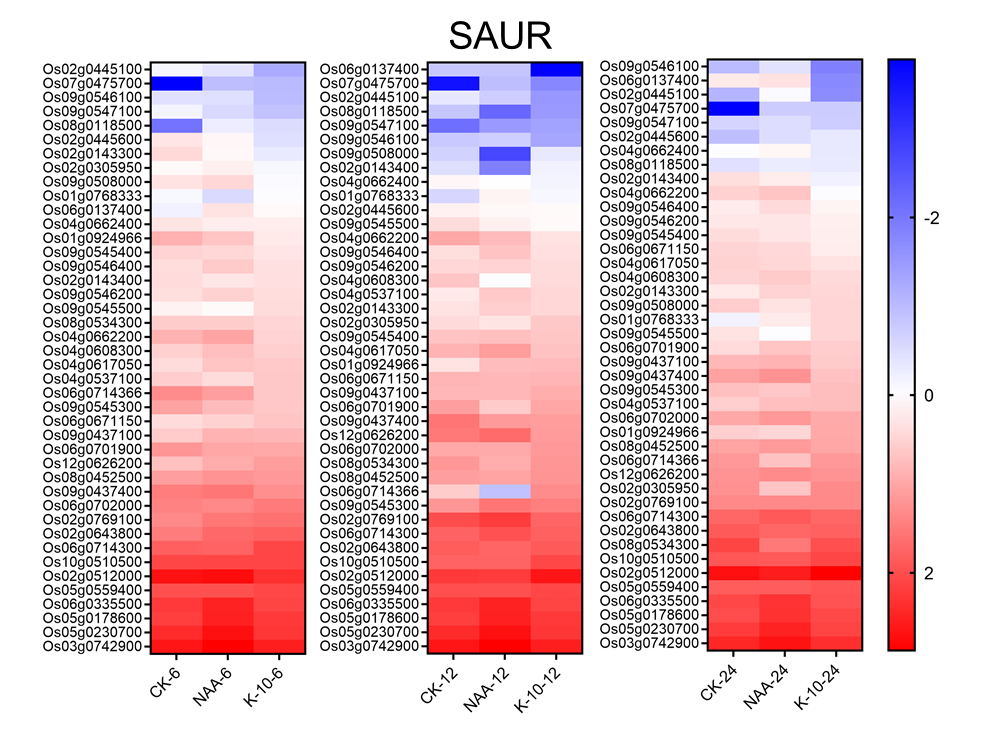


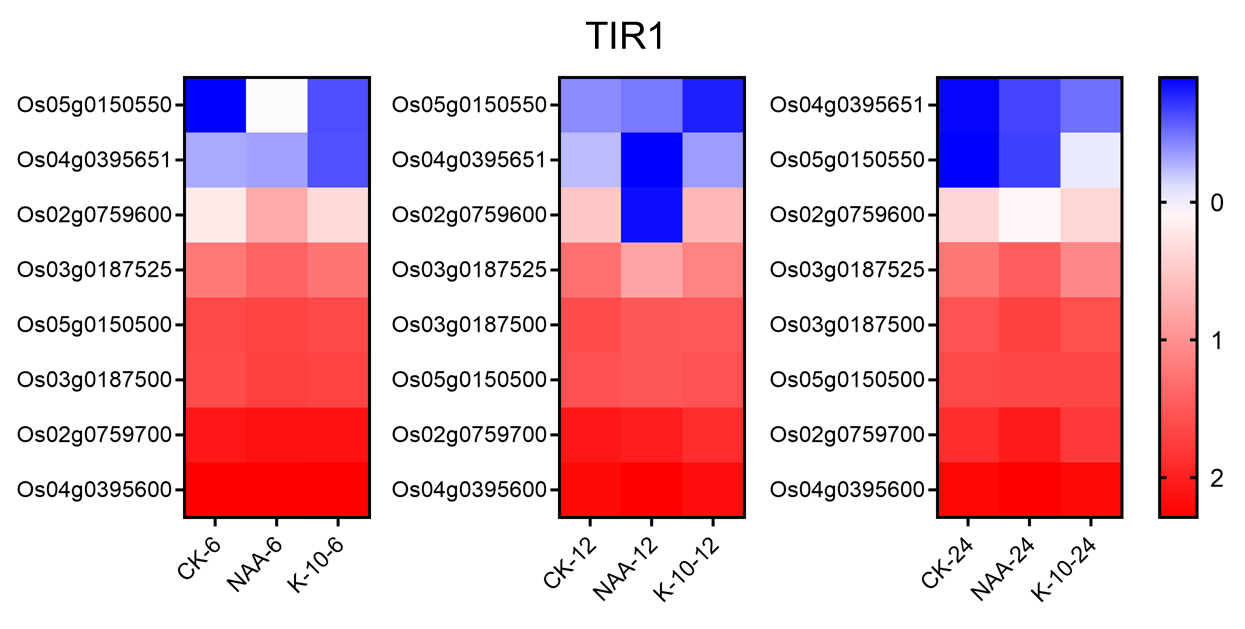


Figure. S4. Heat map of auxin-related genes (*GH3/YUC, LAX, Aux/IAA, SAUR, TIR1*) expression levels in rice roots treated with NAA or K-10 at 6, 12 and 24 h.

(A)
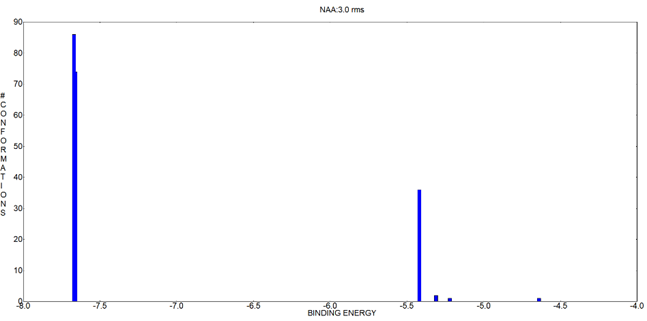


(B)
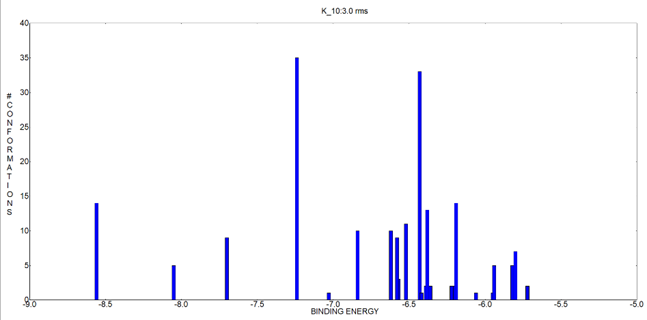


Figure. S5. Cluster of 200 docked conformations for NAA (A) and K-10 (B)

Compound K-1

Compound K-2

Compound K-3

Compound K-4

Compound K-5

Compound K-6

Compound K-7

Compound K-8

Compound K-9

Compound K-10

Compound K-11

Compound K-12

Compound K-13

Compound K-14

Compound K-15

Compound K-16

Compound K-17

Compound K-18

Compound K-19

Compound K-20

Compound K-21

Compound K-22

Figure. S6. The ^1^H NMR and ^13^C NMR spectrum of target compounds

Table S1. The statistical chart of rice hydroponic culture solution

| Chemical element | Concentration（mmol） | Chemical formula | Mass（g/L） |
| --- | --- | --- | --- |
| N | 2.9 | NH_4_NO_3_ | 116.0 |
| P | 0.32 | NaH_2_PO_4_.2H_2_O | 49.9 |
| K | 1 | K_2_SO_4_ | 87.0 |
| Ca | 1 | CaCl_2_ | 111.0 |
| Mg | 1.7 | MgSO_4_.7H_2_O | 418.0 |
| Mn | 0.0091 | MnCl_2_.4H_2_O | 1.802 |
| Mo | 0.00052 | (NH_4_)_6_Mo_7_O_24_.4H_2_O | 0.092 |
| B | 0.018 | H_3_BO_3_ | 1.098 |
| Zn | 0.000015 | ZnSO_4_.7H_2_O | 0.045 |
| Cu | 0.000016 | CuSO_4_.5H_2_O | 0.042 |
| Fe | 0.036 | FeCl_2_.6H_2_O | 9.738 |
| Citric acid monohydrate | | | 14.875 |
